# Web-Based Tools for Text-Based Patient-Provider Communication in Chronic Conditions: Scoping Review

**DOI:** 10.2196/jmir.7987

**Published:** 2017-10-27

**Authors:** Teja Voruganti, Eva Grunfeld, Tutsirai Makuwaza, Jacqueline L Bender

**Affiliations:** ^1^ Institute for Health Policy, Management and Evaluation University of Toronto Toronto, ON Canada; ^2^ Department of Family and Community Medicine University of Toronto Toronto, ON Canada; ^3^ Dalla Lana School of Public Health University of Toronto Toronto, ON Canada; ^4^ Ontario Institute for Cancer Research Toronto, ON Canada; ^5^ ELLICSR Health, Wellness & Cancer Survivorship Centre Department of Supportive Care Princess Margaret Cancer Centre Toronto, ON Canada

**Keywords:** Internet, telemedicine and telecommunication, chronic diseases, physician-patient relations, communication, electronic mail, text messaging, patient portal, patient care team, interdisciplinary communication

## Abstract

**Background:**

Patients with chronic conditions require ongoing care which not only necessitates support from health care providers outside appointments but also self-management. Web-based tools for text-based patient-provider communication, such as secure messaging, allow for sharing of contextual information and personal narrative in a simple accessible medium, empowering patients and enabling their providers to address emerging care needs.

**Objective:**

The objectives of this study were to (1) conduct a systematic search of the published literature and the Internet for Web-based tools for text-based communication between patients and providers; (2) map tool characteristics, their intended use, contexts in which they were used, and by whom; (3) describe the nature of their evaluation; and (4) understand the terminology used to describe the tools.

**Methods:**

We conducted a scoping review using the MEDLINE (Medical Literature Analysis and Retrieval System Online) and EMBASE (Excerpta Medica Database) databases. We summarized information on the characteristics of the tools (structure, functions, and communication paradigm), intended use, context and users, evaluation (study design and outcomes), and terminology. We performed a parallel search of the Internet to compare with tools identified in the published literature.

**Results:**

We identified 54 papers describing 47 unique tools from 13 countries studied in the context of 68 chronic health conditions. The majority of tools (77%, 36/47) had functions in addition to communication (eg, viewable care plan, symptom diary, or tracker). Eight tools (17%, 8/47) were described as allowing patients to communicate with the team or multiple health care providers. Most of the tools were intended to support communication regarding symptom reporting (49%, 23/47), and lifestyle or behavior modification (36%, 17/47). The type of health care providers who used tools to communicate with patients were predominantly allied health professionals of various disciplines (30%, 14/47), nurses (23%, 11/47), and physicians (19%, 9/47), among others. Over half (52%, 25/48) of the tools were evaluated in randomized controlled trials, and 23 tools (48%, 23/48) were evaluated in nonrandomized studies. Terminology of tools varied by intervention type and functionality and did not consistently reflect a theme of communication. The majority of tools found in the Internet search were patient portals from 6 developers; none were found among published articles.

**Conclusions:**

Web-based tools for text-based patient-provider communication were identified from a wide variety of clinical contexts and with varied functionality. Tools were most prevalent in contexts where intended use was self-management. Few tools for team-based communication were found, but this may become increasingly important as chronic disease care becomes more interdisciplinary.

## Introduction

As the number of individuals living with chronic conditions increases [1], the needs of patients are shifting the delivery of health care services based solely on appointments to a patient-driven model that addresses care management and supportive needs on an ongoing basis [2]. This is because the management of chronic diseases often entails a greater degree of patient self-management, supported by a relationship with several providers [3-5].

Numerous organizations such as the Agency for Healthcare Research and Quality and the Institute of Medicine have advocated for the use of electronic health (eHealth) technologies to improve the quality of care, pointing to their value in care coordination and in enabling patients to have greater access to health care providers [6-9]. Especially in the context of chronic or complex conditions, such tools can give patients the opportunity to ask questions, refine understanding, provide updates, and receive test results between appointments. As such, disease self-management may be improved because of timely support from health care providers involved in their care [10,11]. Research has shown that with provider guidance, treatment adherence and motivation to be involved in decision making are improved [12]. Furthermore, although much of the care delivery by health care providers is disease specific or based on medical specialty [9], patients often do not view care in the form of health encounters but rather as continuous between their life and the health care system [13].

Whereas much attention has been paid to tools for telemedicine that allow for patients to upload clinical data (such as glycated hemoglobin [HbA1c] levels or blood pressure values) for the purpose of remote monitoring [14,15], less is known about tools that facilitate dialogue with health care providers. These allow patients to share contextual information, personal narrative, and perspective, which are crucial to the therapeutic function of the patient-provider relationship [16]. Text-based electronic communication, specifically, has grown in popularity because of its simplicity and accessibility [17-19]. This includes formats such as email, phone-based texting, and secure messaging. Furthermore, because communication may be asynchronous (users do not have to be on the Web concurrently), tools for text-based communication have the potential to allow patients to coordinate care across multiple health care providers, in addition to supplementing care provided through appointments [20,21].

As the field of eHealth has rapidly expanded with information and communication technologies (ICTs) taking on a variety of configurations, there is a need for a more focused study on specific forms of eHealth. Recent reviews have broadly examined ICTs in the health care setting for communication between health care providers [22,23] in the pediatric context [24] and the effect on health-related outcomes [10,25-29]. However, such reviews often limited their inclusion to randomized controlled trials (RCTs; which may be inappropriate for eHealth evaluation [30]) and synthesized the effects across several chronic diseases, which may be misleading because such measures are often too heterogeneous to be objectively compared. Furthermore, granularity at the level of features, functions, and implementation of these interventions is often lacking, with studies instead compromising on the depth of description to focus on outcomes [31].

Given the potential value of ICT tools for text-based communication in the health care setting, there is a need to identify and document how common such tools are, what form they take, how they have been used, in what contexts, and for what purpose. Therefore, we undertook a scoping review, as described by Arksey and O’Malley [32], of the published literature and the Internet on Web-based tools for text-based patient-provider communication. The scoping review approach is suitable for reviews that aim to examine the extent, range, and nature of a topic; to identify key concepts in the field; and to identify gaps in the existing literature [33]. Scoping reviews are especially useful when little is known or a field is broad and where a formal systematic review (with narrow selection criteria and focus on study design) may limit what is retrieved. Our specific objectives were to (1) conduct a systematic search of the published literature and the Internet for Web-based tools for text-based communication between patients and physicians; (2) map tool characteristics, their intended use, contexts in which they were used and by whom; (3) describe the nature of their evaluation; and (4) understand the terminology used to describe tools and index articles.

## Methods

### Review Type and Process

We conducted a scoping review using the Arksey and O’Malley framework to identify Web-based tools for text-based patient-provider communication in the published literature and on the Internet [32,34]. We followed the following five steps articulated in this framework: (1) identify the study aim, (2) identify relevant studies, (3) study selection, (4) chart the data, and (5) collate, summarize, and report results [32,33].

### Search of the Published Literature

#### Search Strategy

The search protocols are presented in Multimedia Appendices 1 and ; the Internet search protocol is presented in Multimedia Appendix 3. Given that our target was Internet-connected electronic (Web-based) tools used in health care, we focused our search on MEDLINE (Medical Literature Analysis and Retrieval System Online) and EMBASE (Excerpta Medica Database) for articles in the published literature. The search strategy was developed in consultation with an academic librarian with expertise in eHealth using key concepts, keywords, and controlled vocabulary. We confirmed the completeness of the search strategy by testing it with seed articles that represent expected articles for inclusion [35-38]. We included original studies and captured tools described in editorials and commentaries published up to March 2016. Findings were restricted to those in English because of limited resources for translation services.

#### Selection Criteria

Following the scoping review methodology [32], screening articles for inclusion was done in two stages: title and abstract review and full article review (see Textboxes 1 and 2 for inclusion and exclusion criteria).

**Inclusion criteria.**

Studies were considered for inclusion if they described a tool that:

- Supports Web-based communication between patients and health professionals for within-tool communication (ie, messages sent within the tool are responded to using the tool rather than via phone call outside the tool environment)
- Uses a text-based form of dialogue (including text messages via cell phone)
- Involves communication with patients with one or more chronic conditions, defined as a condition that is ongoing or persistent or requiring complex care, defined as requiring nearly continuous care or otherwise high health care resource utilization and multiple health care providers
- Is used in the health care context
- Is intended for patients and health care providers (physician, nurse, pharmacist, social worker, etc) to communicate regarding direct patient care (defined as private communication about care specific to the patient between health care provider and the patient or surrogate (such as a caregiver), rather than general health advice findable on the open Web. Communication may be guided but not restricted (ie, patient should have the opportunity to ask any question)
- Involves communication between a minimum of one patient and one health care professional (ie, at least two end users)

**Exclusion criteria.**

From the published literature, we excluded:

- Tools that function for information transfer but not communication (eg, lab results, telepathology, telemonitoring of vitals or symptoms [heart rate], and algorithm-based automated feedback)
- Audio or video-based forms of communication that do not include text-based communication
- Electronic medical records, patient health data repositories, and portals that do not have a communication component
- Online support forums, even if they support communication between many patients and many health professionals
- Tools for communication exclusively between patients
- Theoretical or conceptual papers, frameworks, and descriptions
- Offline native apps for mobile devices (ie, those which are not connected to the Internet)
- Tools to support behavior change interventions in otherwise healthy patients (ie, without a chronic condition; eg, smoking cessation, diet, and alcoholism)

#### Study Identification, Selection, and Data Extraction

In the first step of study identification, 2 reviewers (TV and TM) independently reviewed retrieved titles and abstracts from MEDLINE and EMBASE. The reviewers tested agreement on a sample of 100 citations before reviewing all retrieved citations. Where there was uncertainty, citations were included for full article review. We hand-searched the reference lists of identified reviews for potentially relevant articles. We reviewed the full texts of articles designated for inclusion or those labeled as *maybe*. From included articles, 2 authors (TV and TM) independently extracted relevant information. The data extraction form was pilot-tested and revised. It is presented in Multimedia Appendix 4. We extracted data on (1) article characteristics (ie, study setting and disease context); (2) tool characteristics—structure (such as medium of communication), functions (ie, additional components such as viewable care plan), and communication paradigm (ie, one-many, one-one communication flow); (3) intended use, context, and users; (4) evaluation (ie, study design, stage of evaluation, and outcomes); and (5) terminology (ie, tool label or description and medical subject headings [MeSH] terms used to index studies on MEDLINE or keywords on EMBASE). At each step, where there were disagreements, the senior author (JB) was involved to achieve consensus.

### Internet Search

#### Internet Search Strategy

The search of the published literature was supplemented with an Internet search using Google search engine on September 16, 2016 to identify tools that are used but may not have been evaluated or published. Five search queries composed of keywords and Boolean operators were created with the help of the same academic librarian who guided the search of published articles. The first 100 retrieved search results for each query were examined. The same inclusion or exclusion criteria that was used for the published literature was applied to the Google search results, except that findings were not exclusive to tools for chronic diseases because such detail was lacking on most websites.

#### Selection Criteria, Selection Process, and Data Extraction

In the first step, the initial page accessed from the search result was examined (see Multimedia Appendix 3). If it appeared relevant to ICTs or mentioned a tool, it was included. At this stage, search results that led to published primary research articles from an academic database were excluded, as were theoretical or conceptual papers, or those not from the health care context. In the second step, if a search result linked to a specific tool, the website was explored for further information about communication tools that could be used for a patient or caregiver to communicate with a health care provider. Data extraction involved exploring the search result and directly-linked (one-step away) websites for additional information. We modified the data extraction form used for the published literature search to reflect the lack of detail typically available on websites (presented in Multimedia Appendix 5). Two authors (TV and TM) reviewed 20% of search results to establish consistency in extraction, and then the first author (TV) extracted the remaining data.

### Synthesis

Data extracted from published articles and the Internet search were summarized separately. A coding framework was iteratively developed by the reviewing authors (TV and TM) to categorize extracted data according to prespecified definitions based on the published literature or white papers (eg, the Cochrane Collaboration definitions of various study designs [39]), common patterns observed in the data, and expert consultation (JB). The coding framework is presented in Multimedia Appendix 6.

## Results

**Figure 1:**
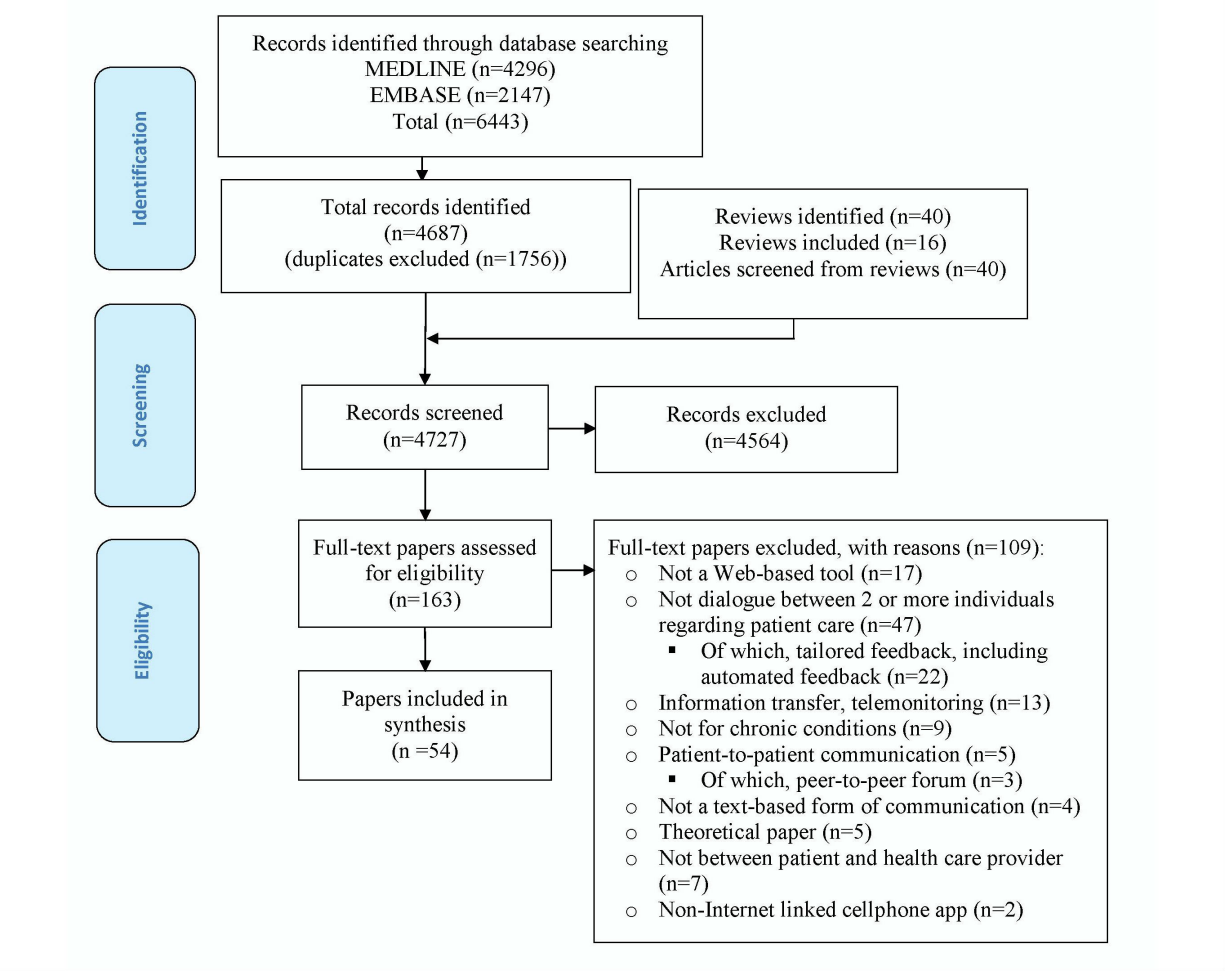
Flow diagram for published literature search.

**Figure 2:**
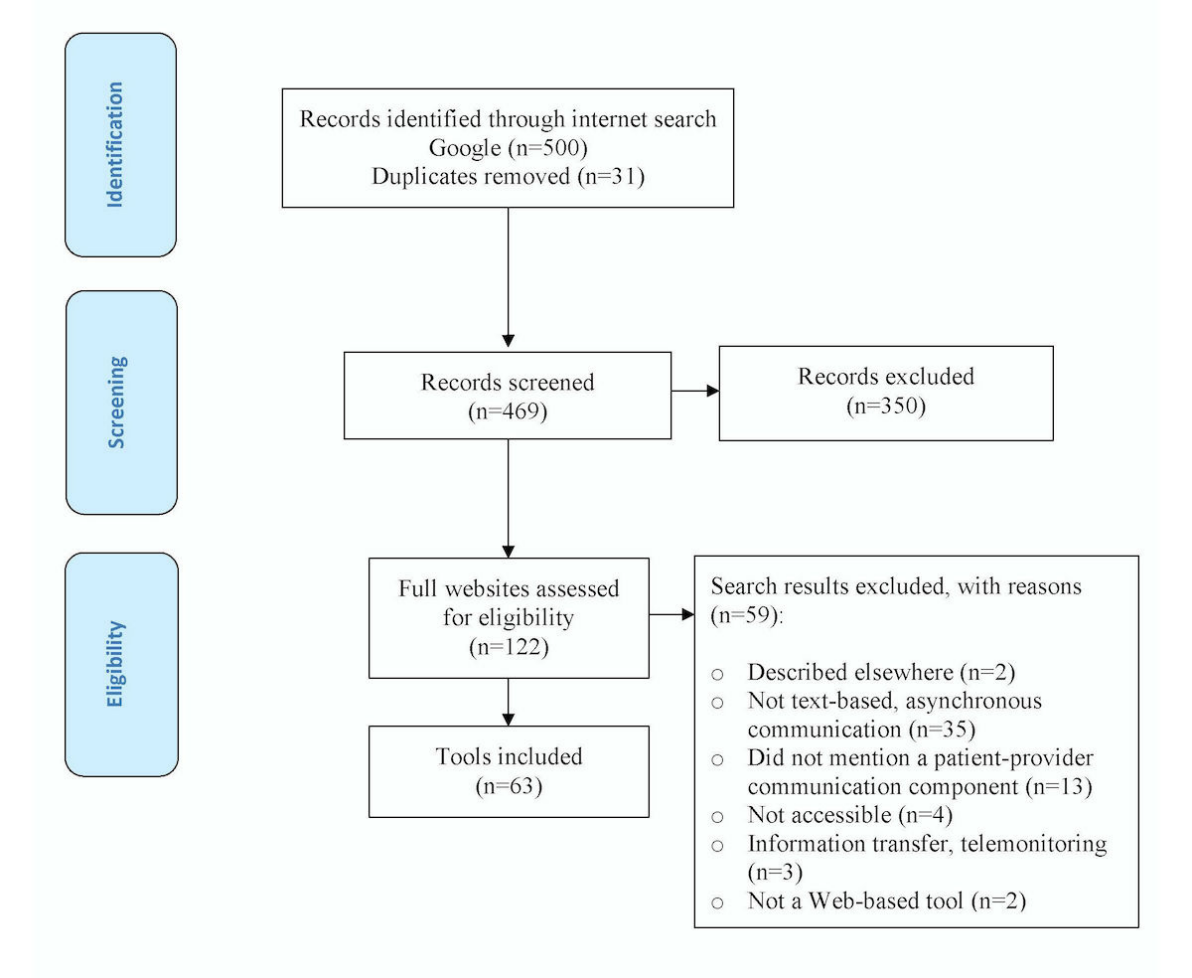
Flow diagram for Internet-based search.

The search of published literature retrieved 6443 results from MEDLINE (n=4296) and EMBASE (n=2147). After removal of duplicates (n=1756), 4687 titles and abstracts were screened, 121 full-text articles were reviewed, and 40 articles met the selection criteria. At the screening stage, chance-corrected agreement between the 2 reviewers was 0.51 (95% CI 0.44-0.57), calculated with Cohen's kappa, and raw agreement was 0.97. Additionally, 40 review articles were identified, of which 16 were hand-searched for articles meeting the selection criteria. Fourteen studies from the review articles met our selection criteria, bringing the total number of studies included to 54 (see Figures 1 and 2).

### Article Characteristics

**Figure 3:**
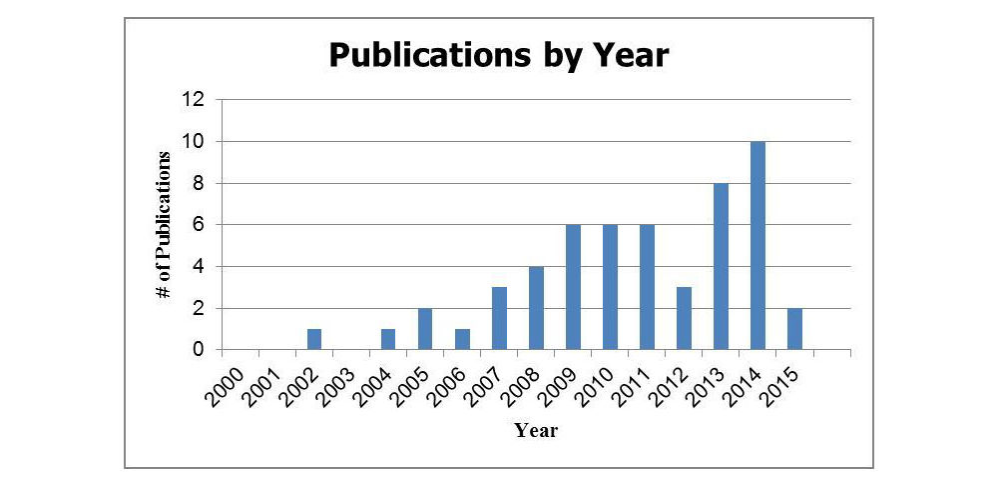
Published articles by year (n=54).

Of the 54 articles, there were 53 unique studies describing 47 unique tools (after accounting for multiple articles from one study). The earliest article identified was published in 2002. As shown in Figure 3, the number of published articles on this topic has been increasing annually. The majority of articles were from the United States (48%, 26/54; see Table 1). Most studies were conducted at tertiary care outpatient clinics specializing in a particular condition (51%, 27/53), though a large number were from the primary care setting (36%, 19/53). Only four studies (7.5%, 4/53) were conducted in exclusively pediatric populations (<18 years old).

### Tool Characteristics

Characteristics of tools from published articles were organized according to tool structures, functions, and communication paradigm and are presented in full in Table 2 (by characteristic) and Multimedia Appendix 7.

#### Structures

Of the 47 tools identified, the majority (74.5%, 35/47) were Internet-enabled applications accessible from a Web browser, whereas 9 (19%, 9/47) were native applications developed as computer software or for use on a mobile phone. Most (77%, 36/47) were multidimensional tools with multiple features and functions, of which 30% (14/47) were part of an informational or educational website, and 40% (19/47) were patient portals; 30% (14/47) were stand-alone communication tools.

**Table 1:** Published article characteristics (n=54).

Characteristic		n (%)
**Publication country of origin (n=54**)		
	Australia	1 (2)
	Canada	5 (9)
	China	1 (2)
	Finland	2 (4)
	Germany	1 (2)
	Netherlands	4 (7)
	Norway	7 (13)
	Portugal	1 (2)
	Slovenia	1 (2)
	Spain	2 (4)
	Sweden	2 (4)
	Switzerland	1 (2)
	United States	26 (48)
**Unique studies (n=53)**		
	Original study	48 (91)
	Protocol	4 (7.5)
	Editorial or commentary	1 (2)
**Study context or setting of use (n=53)**		
	Academic (ie, Department of behavioral sciences)	4 (7.5)
	Business (ie, CVS and Walmart)	1 (2)
	Integrated health care organization (ie, Kaiser Permanente)	2 (4)
	Primary care	19 (36)
	Tertiary care outpatient clinics	27 (51)
**Population (n=53)**		
	Adults or all	49 (92)
	Pediatrics (<18 years)	4 (7.5)
**Disease or clinical area of interest (n=68)^a^**		
	Cardiovascular disease or stroke	6 (9)
	Chronic respiratory condition	10 (15)
	Diabetes	20 (29)
	Mental health	8 (12)
	Chronic pain	5 (7)
**Other**		
	Dermatology	2 (3)
	Irritable bowel disease or syndrome	2 (3)
	Cerebral palsy	1 (1.5)
	Human immunodeficiency virus (HIV) or acquired immunodeficiency syndrome (AIDS)	1 (1.5)
	Rheumatic disease	1 (1.5)
	Obesity	1 (1.5)
	Hypertension	2 (3)
	Fibromyalgia	2 (3)
	Cystic fibrosis	1 (1.5)
	Impaired mobility	1 (1.5)
	Nonspecific (*Chronically ill*)	5 (7)

^a^Some studies evaluated the tool in multiple contexts, for example, in diabetes and mental health.

#### Functions

Two categories of communication functions were identified: unstructured and structured text-based communication. The majority of tools (79%, 37/47) involved unstructured text-based communication that allowed a patient to enter open-ended free text. Conversely, 10 tools involved structured communication whereby a patient would submit an inquiry into a form with structured fields that returned a response to questions (tools with automated responses were excluded). The majority of tools (77%, 36/47) had other functions in addition to the communication component, including disease information or education (53%, 25/47), symptom diary or tracker (45%, 21/47), and viewable care or treatment plans (25.5%, 12/47).

#### Communication Paradigm

The majority of tools (94%, 44/47) used asynchronous communication of which two specified that health care providers were to respond in a specified amount of time (ie, within 3 days). With most tools (83%, 39/47), patients could communicate with one specific health care provider (ie, one-to-one communication). Only 17% (8/47) of tools were described as allowing the patient to communicate with their health care team or multiple health care providers (ie, one-to-many communication). These were evaluated in the diabetes (3/8), respiratory conditions (1/8), human immunodeficiency virus (1/8), depression (1/8), and general outpatient (1/8) contexts. One tool described having patient-professional and interprofessional communication paradigms in patients with cerebral palsy. Eighteen tools (38%, 18/47) described allowing the patient to communicate with their *own provider* (presumably, someone involved in their direct care).

#### Intended Use, Context, and Users

The intended use of tools described in articles were grouped into four categories: symptom reporting (49%, 23/47), lifestyle or behavior modification (36%, 17/47), care planning (4%, 2/47), and medication adherence (2%, 1/47). No intended use was stated in the articles for 4 tools though these were in nonrandomized studies where the tool was not evaluated as an intervention.

Studies were conducted in several different chronic disease populations, with many studies evaluating tools in multiple disease contexts. In total, the studies covered 68 health conditions. Notably, 29% (20/68) were evaluated for diabetes, 15% (10/68) for chronic respiratory conditions, and 12% (8/68) for mental health. Few studies were evaluated in cardiovascular disease (CVD; 9%, 6/68).

The type of health care provider who used the tool varied greatly: 23% (11/47) were used by nurses, 19% (9/47) by physicians, and 30% (14/47) involved allied health professionals of various disciplines (see Table 2 and Multimedia Appendix 7 for details). Only two studies mentioned that providers were given monetary compensation for tool use.

**Table 2:** Tool characteristics, intended use, and users (n=47). The table classifies variables according to unique tools rather than individual studies as the unit of analysis.

Characteristic			n (%)
**Structures**			
	**Medium of communication or format**		
		Web-based tool	35 (4.5)
		Hybrid Web and software application	6 (13)
		Mobile phone–based native app (ie, short message service)	3 (6)
		Email-based application	3 (6)
	**Component of another platform**		
		Patient portal	19 (40)
		Informational or educational website	14 (30)
		Stand-alone	14 (30)
**Functions**			
	**Type of communication**		
		Unstructured communication (patient-provider free form dialogue)	37 (79)
		Structured communication (tailored feedback)	10 (21)
	**Number of tools with functions beyond patient-provider communication component**		36 (77)
		With 3 or more additional functions	19 (40)
		Linked to a health record	9 (19)
		Linked to laboratory or test results	12 (25.5)
		Linked to appointment or scheduling	7 (15)
		Linked to viewable care or treatment plan	12 (25.5)
		Linked to new prescription requests	3 (6)
		Linked to prescription renewal	8 (17)
		Linked to symptom diary or tracker	21 (45)
		Linked to disease information or education	25 (53)
**Communication paradigm**			
	**Asynchronous tools**		
		Asynchronous	44 (94)
		Of asynchronous tools, time-limited (response from provider within a specified time window)	2 (4)
		Synchronous	2 (4)
		Both	1 (2)
	**Patient-provider communication flow**		
		One-many	8 (17)
		Communication with own provider	4 (50)
		One-one health care provider	39 (83)
		Communication with own provider	18 (46)
	**Of patient-multiple provider tools (n=8), direct communication with each member of health care team (providers receive information at the same time)**		
		Yes	1 (12.5)
		No	3 (37.5)
		Unclear	4 (50)
			
**Intended use and users**			
	**Intended use of communication intervention^a^**		
		Lifestyle or behavior modification	17 (36)
		Symptom reporting	23 (49)
		Care planning	2 (4)
		Medication adherence	1 (2)
		Not specified	4 (8.5)
	**Type of health care provider intended to use tool with patients or caregivers**		
		Nurse	11 (23)
		Physician	9 (19)
		One of several professions (ie, physician or nurse or social worker)	7 (15)
		Case manager or social worker	5 (11)
		Psychologist	4 (8.5)
		Therapist or counselor	4 (8.5)
		Pharmacist	1 (2)
		Research assistant	1 (2)
		Not specified	5 (11)
**Other**			
	**Compensation to health care providers**		
		Did not provide compensation	45 (96)
		Did provide compensation	2 (4)
	**Tool access**		
		Free through research participation	29 (62)
		Organizational license	10 (21)
		Prior registration required via website or service	8 (17)
	**URL available in article**		
		Yes	17 (36)
		No	30 (64)

^a^Purposes are grouped based on descriptions from each paper.

### Evaluation Characteristics

#### Study Design and Study Stage

The evaluation characteristics of completed studies (ie, excluding protocols) are reported in Table 3. Twenty-five studies were RCTs. Twenty-three were nonrandomized studies, of which nine were prospective cohort studies, four were retrospective cohort studies, four were quasi-experimental or non-RCTs, two were cross-sectional studies, one was a cost-effectiveness study, and three were qualitative studies. All were real-world evaluations and not in a laboratory setting. Regarding the stage of study according to the 2008 MRC Framework for the Evaluation of Complex Interventions [40], 96% (24/25) of RCTs were at the evaluation stage compared with 26% (6/23) of nonrandomized studies, of which 43.5% (10/23) were at the feasibility and piloting stage. The only studies at the implementation stage were nonrandomized studies (30%, 7/23). The sample size of RCTs ranged from 15 to 415 patients and spanned 1 to 20 months of follow-up. By comparison, the sample size of nonrandomized studies ranged from 2 in a stand-alone qualitative study to 14,102 in a retrospective analysis of administrative cohort data.

#### Study Outcomes

See Table 3 for outcomes captured: RCTs (n=37 outcomes measured) tended to focus mostly on clinical outcomes (70%, 26/37; eg, cholesterol reduction, depression symptoms, and patient activation), whereas nonrandomized studies (n=35 outcomes measured) examined outcomes related to acceptability (11%, 4/35), feasibility (9%, 3/35), and usability (14%, 5/35) more often. Experience-related outcomes (eg, perceptions and open-ended feedback) were not captured in RCTs; however, they were captured in nonrandomized studies either as stand-alone qualitative studies (9%, 3/35) or as part of a study capturing quantitative and qualitative outcomes (9%, 3/35). 

**Table 3:** Evaluation characteristics of unique completed studies (n=48). It refers to unique studies, counting studies resulting in multiple publications and excludes protocols, editorials, or commentaries.

Study design and evaluation characteristics			Outcome
**Randomized controlled trials (n=25)**			
	Is the communication component the primary feature or a supplemental feature? (n)		Primary feature=17
			Supplemental feature=8
	Stage of study^a^, n		Development=0
			Feasibility and piloting=1
			Evaluation=24
			Implementation=0
	Type of results captured in each study^b^, n		Acceptability=1
			Clinical=26
			Usability=2
			Feasibility=1
			Usage=7
	Sample size, median (IQR; range)		104 (75.5-140; 15-415)
	Study length of follow-up in months, median (IQR; range)		8 (3-12; 1-20)
**Nonrandomized studies (n=23)**			
	**Prospective cohort studies (n=9)**		
		Is the communication component the primary feature or a supplemental feature? (n)	Primary feature=7
			Supplemental feature=2
		Stage of study^a^, n	Development=0
			Feasibility and piloting=7
			Evaluation=2
			Implementation=0
		Type of results captured in each study^b^, n	Acceptability=2
			Clinical=6
			Experience^d^=3
			Feasibility=2
			Usability=4
			Usage=1
		Sample size, median (IQR; range)	21 (15-30; 6-222)
		Study length of follow-up in months, median (IQR; range)	6 (3-6.5; 1-13)
	**Retrospective cohort studies (n=4)**		
		Is the communication component the primary feature or a supplemental feature? (n)	Primary feature=1
			Supplemental feature=3
		Stage of study^a^, n	Development=0
			Feasibility and piloting=0
			Evaluation=1
			Implementation=3
		Type of results captured in each study^b^, n	Clinical=2
			Usage=3
		Sample size, median (IQR; range)	2603 (1750.75-5718.5; 157-14102)
		Study length of follow-up in months, median (IQR; range)	13.5 (10.5-17.25; 6-24)
	**Quasi-experimental/nonrandomized controlled trials (n=4)**		
		Is the communication component the primary feature or a supplemental feature? (n)	Primary feature=4
			Supplemental feature=0
		Stage of study^a^, n	Development=0
			Feasibility and piloting=1
			Evaluation=3
			Implementation=0
		Type of results captured in each study^b^, n	Acceptability=1
			Clinical=3
			Usage=1
		Sample size, median (IQR;range)	141 (93.25-348.75;46-876)
		Study length of follow-up in months, median (IQR; range)	9 (6-14.5; 6-22)
	**Cross-sectional surveys (n=2)**		
		Is the communication component the primary feature or a supplemental feature? (n)	Primary feature=2
			Supplemental feature=0
		Stage of study^a^, n	Development=0
			Feasibility and piloting=0
			Evaluation=0
			Implementation=2
		Type of results captured in each study^b^, n	Acceptability=1
			Feasibility=1
			Usability=1
		Sample size, median (IQR; range)	2327.5 (1236.25-3418.75; 145-4510)
		Study length of follow-up in months, median (IQR;range)	N/A^e^
	**Cost-effectiveness analyses (n=1)**		
		Is the communication component the primary feature or a supplemental feature? (n)	Primary feature=1
			Supplemental feature=0
		Stage of study^a^, n	Feasibility and piloting=0
			Evaluation=0
			Implementation=1
			Development=0
		Type of results captured in each study^b^, n	Costs or clinical=1
		Sample size, median (IQR; range)	778
		Study length of follow-up in months, median (IQR; range)	12 months
	**Qualitative studies (n=3)**		
		Is the communication component the primary feature or a supplemental feature? (n)	Primary feature=2
			Supplemental feature=1
		Stage of study^a^, n	Development=0
			Feasibility and piloting=2
			Evaluation=0
			Implementation=1
		Type of results captured in each study^b^, n	Experience^c^=3
		Sample size–median (IQR; range)	7 (4.5-23; 2-39)
		Follow-up (yes), n	2
		Study length of follow-up in months, median (IQR; range)	3 (2-4; 1-5)

^a^Definitions according to 2008 MRC Framework for Evaluation of Complex Interventions. See coding framework for elaboration.

^b^All types of results (outcomes) in a study are counted so that multiple outcomes may be counted from individual studies.

^c^Three studies captured qualitative results as secondary outcomes. Three studies were stand-alone qualitative studies.

^d^N/A: not applicable.

### Terminology

The terminology used to describe the tools was explored in published articles by examining author descriptions of the tool and the terms used to index the articles by academic librarians. “Portal” was often used to describe tools with more than three additional functions (42%, 8/19). Of studies where the communication component was the primary feature, “Web-based” (29%, 7/24) and “Internet-based” (21%, 5/24) were frequently used as adjectives in intervention descriptions. However, the actual intervention descriptor varied considerably (ie, diaries, self-management intervention). Regarding the indexing terminology of articles, the MeSH terms Internet (n=40), telemedicine and telecommunication (n=11), physician-patient relations (n=12), cell phones (n=9), communication (n=7), electronic health records (n=7), and electronic mail (n=7) and therapy, and computer-assisted (n=5) appeared 5 or more times.

### Internet Search Results

An Internet search identified websites for 63 unique tools, 82.5% (52/63) of which were identified from health care institution websites (hospitals and care networks) and 17.5% from businesses (including tool developer companies; see Table 4). None of the tools identified on the Internet were found in the published literature. The majority of health care institution–based tools came from 6 developers or companies: FollowMyHealth (19%, 10/52), Athena Health (15%, 8/52), MyChart Epic Systems (15%, 8/52), eClinicalWorks (11.5%, 6/52), NextGen Healthcare Information Systems LLC (10%, 5/52), and Cerner IQ Health (8%, 4/52). Most (94%, 59/63) of the websites described their tool as having a communication component integrated with an electronic health record (EHR). Most websites (84%, 53/63) also reported that their tool allowed the patient to communicate with one health care provider, 11 (17.5%, 11/63) of which stated in the description that patients could talk with their *own provider* directly. Two websites described tools that allowed patients to talk with multiple health care providers. Of 60 tools (95%, 60/63) that used asynchronous text-based communication, only 8 (13%, 8/63) of the websites stated that a response from a provider could be expected within a specified time frame (ie, 3-5 days).

**Table 4:** Tools identified from the Internet search (n=63).

Characteristic		n (%)
**Organization type**		
	Health care institution (ie, hospitals and care networks)	52 (82.5)
	Business (ie, tool developers)	11 (17.5)
**Health record integration**		
	Yes	59 (94)
	No	3 (5)
	Unclear	1 (2)
**Target population**		
	Outpatients	47 (75)
	Both	8 (13)
	Not specified or unclear	6 (9.5)
**Health care provider intended to use tool with patients or caregivers as described (excluding business tools)**		
	“Members of the health care team”	11 (17.5)
	“Doctor's office”	18 (29)
	“Physician”	5 (8)
	“Nurse”	2 (3)
	“Provider”	11 (17.5)
	Unclear	4 (6)
**Asynchronous tools**		
	Asynchronous	60 (95)
	Of asynchronous tools, time-limited (response from provider within a specified time window)	8 (13)
	Unclear	3 (5)
	Synchronous	0 (0)
**Patient-provider communication flow**		
	One-many	2 (3)
	Communication with own provider	0 (0)
	One-one	53 (84)
	Communication with own provider	11 (17.5)
	Unclear	8 (13)
**Product names of health care institution tools (n=52)**		
	Athena Health	8 (15)
	Beth Israel Deaconess Medical Center	1 (2)
	Carolinas Healthcare	1 (2)
	Cerner IQ Health	4 (8)
	eClinicalWorks	6 (11.5)
	FollowMyHealth	10 (19)
	IASIS Healthcare	1 (2)
	Intermountain Healthcare	1 (2)
	MedFusion-Greenway Health	1 (2)
	MyChart Epic Systems	8 (15)
	NextGen Healthcare Information Systems LLC	5 (10)
	Partners HealthCare	1 (2)
	RelayHealth	2 (4)
	University of Wisconsin-Madison	1 (2)

## Discussion

### Principal Findings

In this scoping review, we found 54 published articles that described text-based patient-provider communication tools for chronic diseases. These tools were predominantly accessed from websites as opposed to Internet-linked native apps and mainly functioned as part of a multifunction platform such as patient-facing portals. Few tools enabled patients to communicate with multiple health care providers at the same time (ie, one-to-many communication). Tools were used for lifestyle or behavior modification, symptom reporting, care planning, and medication adherence purposes. We found that the majority of tools were studied in the diabetes and chronic respiratory condition contexts. Around half of the studies were RCTs that focused on clinical outcome evaluations, whereas nonrandomized studies examined impact on outcomes such as acceptability and usability. Terminology used to describe the tools varied greatly by intervention type and functionality and did not consistently include the theme of communication. The Internet search results did not show overlap with tools found in the search of published articles, and tools found on the Internet were primarily produced by a small number of developers.

We found many tools that facilitated both communication and sharing of data. Most studies (77%, 36/47) described tools with capabilities additional to communication such as access to EHRs (25% 9/36), lab or test results (33%, 12/36), and care or treatment plans (33%, 12/36), among others. Due to the shared infrastructure, platforms for communication can easily accommodate components for information sharing (eg, lab test results) to allow for more productive interaction. Building on Wagner’s Chronic Care Model [41] which delineates organizational domains needed to support patient self-management and interaction with the health care team, the eHealth Enhanced Chronic Care Model (eCCM) by Gee et al [42] reenvisions chronic care management as reinforced by the breadth of eHealth technologies. The eCCM postulates that the sharing of data and information in different ways, which is facilitated by technologies, can enhance patient and provider knowledge and wisdom, making communication between patients and health care teams more productive. Therefore, multifunction platforms may make communication more informed through added access to medical data.

The growing recognition that care of chronic conditions is rooted in self-management has also been met with a parallel shift in the role of health care providers from experts to collaborators with patients [11]. We identified 8 tools that allowed patients to communicate with multiple health care providers or their *team* as a group (ie, one-to-many communication). Only one tool [43] clearly described that it facilitated patient-professional and interprofessional communication. Intervention descriptions of other studies were vague as to whether patient messages were simultaneously delivered to all team members or to a moderator who triaged messages to health care providers. Here, we found that nurses were most often the provider who used the communication tool with patients (23%, 11/47). Also, 15% (7/47) of tools were described as involving patient communication with individuals of one of several different professions (ie, a nurse, physician, or social worker) suggesting that patients are not necessarily in direct contact with their own physician. The importance of patient-multiple provider tools may be magnified in contexts where multiple providers are responsible for different aspects of care and where provider decisions can benefit from the insight of other providers. Tools for collaboration are not novel [44]; in business, collaborative platforms such as Microsoft’s Yammer, Slack, and Hipchat, which facilitate synchronous (such as live video), asynchronous individual and group-based communication, and data exchange with multiple users are prevalent [45]. Traditionally, responsibility for patient care transfers from physician to physician according to disease or treatment modality, and therefore tools for asynchronous collaborative communication may be better suited in health care [46]. However, lack of financial compensation for physician consults (including group-based interactions) and concerns about security of data are significant barriers to the use of ICTs for physicians to communicate with each other about a case and with the patient directly [47] and may partly explain the dearth of tools for teams. Only two articles [48,49] identified here from the literature and none from the Internet-based search mentioned compensation for health care provider tool use.

Our findings indicate that the number of studies of patient-physician text-based communication tools has increased in the last decade for purposes related to self-management and for many conditions, pointing to the broadening appeal of this communication medium. We found that tools for certain chronic conditions with high prevalence were most common (diabetes=20 and chronic respiratory conditions=10) but found few tools for several less common conditions (eg, cerebral palsy and cystic fibrosis). Notably, we found very few tools for other common chronic conditions such as CVD (n=6) and none for cancer. This pattern could be reflective of the type of care associated with these conditions: for typical cases of diabetes [50] and respiratory conditions such as asthma [51], care protocols usually emphasize supported self-management. Furthermore, conditions such as diabetes and respiratory diseases are particularly costly, with progression to advanced stage or complications, such that prevention and management at early stages is viewed as an effective approach [52-54]. Though CVD also entails a degree of self-management, our findings could suggest that dialogue with a provider is less necessary. Instead, it can be substituted with telemonitoring (eg, cardiac telemetry and blood pressure monitoring), which are part of usual CVD care [52,53]. These conditions also make use of specialized diagnostic and treatment protocols that involve different professions. As such, these conditions may benefit from tools allowing for patient-multiple provider communication to address complex needs. However, none of the patient-multiple provider communication tools found here were from the CVD and cancer contexts and could suggest a potential gap.

Evaluations of effect of the identified tools tended to adopt RCT designs (n=25) where outcomes were clinical, whereas non-RCTs were more inclusive in capturing implementation outcomes. We found, however, that usage data were poorly reported across studies of all designs. Usage data, as a measure of process, are critical to understanding why an intervention has functioned in a particular context, as the data provide insight into which components of an intervention were used and may be responsible for the observed effect [27]. It is therefore important for appreciating the generalizability of findings in other contexts. Traditional study designs, such as RCTs, may not adequately address the dual goals of unbiasedly ascertaining effect and sufficiently capturing the practical realities of implementation [54]. Furthermore, we encountered few qualitative studies (6%, 3/53) and mixed-methods studies (11%, 6/53), which are better suited to understanding how the users, setting, and cointerventions in the existing environment have affected the intervention [55]. Novel designs, for example, hybrid trials [56] for evaluating complex interventions such as eHealth tools, incorporate clinical and process evaluations to better contextualize findings and shed light on the mechanisms of action.

The terminology used to describe eHealth tools presented a challenge for conducting a review on this topic because of the diversity of terms and the lack of standardized vocabulary to label them. We found that the theme of communication was not always reflected in descriptions or indexing terminology. Multifunction tools were often described as portals, whereas other tools made use of technology-related adjectives added onto standard intervention terms (eg, Web-based self-help and e-coaching). Articles were sometimes indexed with MeSH terms that denoted specific functions such as “Patient-physician relations” and “Therapy, Computer-assisted” or with recognized communication modalities such as “Electronic Mail” and “Cell Phones.” MeSH terms for narrower descriptions such as “Secure Messaging” are lacking, although “Patient Portal” was newly introduced in July 2016. These patterns reflect the inchoate and rapidly evolving nature of this field, may indicate that structured taxonomies in eHealth are yet premature, and suggest that ontologies relating to the terminology of similar interventions may be needed to facilitate article retrieval. These findings are also suggestive of the trade-off in performing searches between the need for sensitivity to accurately detect articles on interventions of common functionality but varied design, and specificity of labeling articles with descriptions that are transparent and replicable. As noted elsewhere about reviewing complex interventions, overall, keywords should attempt to reflect both breadth and depth to maximize capture [57].

In performing a parallel search of the Internet, we found that most tools were developed by six health care software companies. This may speak to the greater Internet visibility of those tools produced by companies with the biggest market share. None of the tools found on the Internet were found in the published literature search (or vice versa). This could suggest that many commercially available tools bypass rigorous, research-driven evaluation (or research findings are not shared) in the process of creating a product whose goal is to meet demand rather than understand improvement in health outcomes [58]. However, the compromise is that without research to bolster the theoretical or evidentiary rationale of such products, they may not meet effectiveness goals. Conversely, tools evaluated and published in articles were not found publically on the Internet, suggesting that research-driven tools often lack the support needed for iterative development and long-term sustainability if they do not have a commercial or business-driven foundation.

### Limitations

The scoping review methodology appropriately pursues breadth in identifying articles with a trade-off to performing an in-depth study of specific literature. Although we aimed to conduct a comprehensive search with an extensive search strategy (using 159 technology-related terms), it is possible that we may have missed some relevant articles, given the lack of standardized terminology in this field. We limited our search to MEDLINE and EMBASE because our objectives were related to health and also because we found few articles of relevance in other databases (eg, Cumulative Index to Nursing and Allied Health Literature or CINAHL) while developing the search strategy. Through screening and selection, we did not find tools implemented in the cancer context. Whereas cancer is considered a chronic disease by organizations such as the World Health Organization, it is possible that medical databases have only recently begun to index cancer-related articles within terms such as chronic diseases (we did not base our search protocol on named chronic diseases, as that would have limited the contexts in which tools are found.). Regarding the Internet search, we acknowledge that, as the Google search engine algorithms are continuously updated, it is unlikely that the Internet search is replicable. However, the purpose of the Internet search in this study was to complement and compare with findings from the published literature rather than report stand-alone results. We limited our review to English-language publications. However, given the large number of findings from countries with a primary language other than English (eg, Norway and The Netherlands), we may have missed publications that have not been translated or are not accessible from databases.

### Conclusions

We conducted a scoping review of Web-based tools for text-based patient-physician communication. In this review, we identified tools for a variety of chronic conditions, the majority of which targeted diabetes and chronic respiratory conditions for the purposes of updating providers about symptoms or for providers to facilitate lifestyle or behavior change. Our findings seem to suggest that asynchronous text-based patient-provider communication is increasingly being used to support patient self-management functions for conditions such as diabetes, which, when properly controlled, are amenable to routine online check-ins. On the other hand, we identified few tools for CVD, which could suggest a gap in the literature. We found that there were few tools for patient-multiple provider communication, which will become a growing area of interest to patients, providers, developers, and organizers of care as care for chronic conditions becomes more interdisciplinary. The terminology used to describe tools and index articles is widely varied, suggesting that to optimize findability, researchers need to label articles by both tool characteristics and communication functionality. Reviewers may still need to cast a wide net to capture potentially relevant tools, and our findings suggest a need for ontologies that associate similar terms of related interventions to improve article retrieval without diluting the specificity with which authors describe tools. The difference in findings between the search of the published literature and the Internet could reflect the competing need for rigorous evaluation and for real-world implementation to both generate revenue for sustainability and upgrades of tools over time. In an era of health care where patients expect information on demand, the provision of information supplemented by communication with their providers can enable care when and where a patient needs it, contributing to the betterment of chronic disease management.

## Appendix

Multimedia Appendix 1 MEDLINE(R) (Ovid Interface) 1946- Week 1 March 2016.

Multimedia Appendix 2 EMBASE (Ovid Interface) 1946- Week 1 March 2016.

Multimedia Appendix 3 Google search strategy.

Multimedia Appendix 4 Data extraction form for published literature search.

Multimedia Appendix 5 Internet data extraction form.

Multimedia Appendix 6 Coding framework.

Multimedia Appendix 7 References by selected tool design and function.

## Abbreviations

CVD: cardiovascular disease
eCCM: eHealth Enhanced Chronic Care Model
eHealth: electronic health
EHR: electronic health record
EMBASE: Excerpta Medica Database
ICT: information and communication technology
MEDLINE: Medical Literature Analysis and Retrieval System OnlineMeSH: medical subject heading
RCT: randomized controlled trial
