# Supplementary material for: Web-Based Tools for Text-Based Patient-Provider Communication in Chronic Conditions: Scoping Review
Source: J Med Internet Res. 2017 Oct 27;19(10):e366. doi: 10.2196/jmir.7987 (PMC5681721; doi:10.2196/jmir.7987)
Supplement: Multimedia Appendix 1 [file jmir_v19i10e366_app1.pdf]

Appendix 1 MEDLINE(R) (Ovid Interface) 1946- Week 1 March 2016

1	exp internet/
2	internet:.mp.
3	exp informatics/
4	exp computer-assisted instruction/
5	online.mp.
6	on-line.mp.
7	(virtual not virtual realit:).mp.
8	world wide web:.mp.
9	worldwide web:.mp.
10	www.tw.
11	web.tw,kw.
12	web page:.mp.
13	webpage:.mp.
14	web site:.mp.
15	website:.mp.
16	exp computer communication networks/
17	(portal? and (internet* or online or on-line or computer* or electronic or web or webbased or telehealth or tele-health)).mp.
18	(portal?? adj3 patient??).mp,kw.
19	ehealth.mp.
20	e-health.mp.
21	semantic web?.mp.
22	blog:.mp.
23	folksonom:.mp.
24	mashup:.mp.
25	pod cast:.mp.
26	podcast:.mp.
27	(social adj2 bookmark:).mp.
28	(social adj2 book-mark:).mp.
29	(social adj2 software:).mp.
30	(sociable adj2 technolog:).mp.
31	(social adj2 technolog:).mp.
32	tag cloud:.mp.
33	(virtual adj2 collabor:).mp.
34	web api:.mp.

35 (web adj2 syndicat:).mp.
36 webcast:.mp.
37 web-cast:.mp.
38 web-log:.mp.
39 weblog:.mp.
40 wiki:.mp.
41 social network:.mp.
42 (social adj2 utilit:).mp.
43 chat.mp.
44 chatroom*.mp.
45 chat-room*.mp.
46 chat group*.mp.
47 chatgroup*.mp.
48 chat techno*.mp.
49 meebo.mp.
50 "second life".mp.
51 secondlife.mp.
52 uhealth.mp.
53 (ubiquit* adj2 comput*).mp.
54 "u-comput*".mp.
55 patientslikeme*.mp.
56 "www.patientslikeme.com".mp.
57 (digital adj2 divid*).mp.
58 (digital adj2 inequit*).mp.
59 exp information systems/
60 exp computer systems/
61 exp telecommunications/
62 exp user-computer interface/
63 exp computer literacy/
64 exp attitude to computers/
65 "u-health*".mp.
66 "e-health*".mp.
67 twitter.mp.
68 tweet.mp.
69 epatient*.mp.
70 "e-patient*".mp.
71 edoctor*.mp.

72 e-doctor*.mp.
73 ephysician*.mp.
74 "e-physician*".mp.
75 elearn*.mp.
76 "e-learn*".mp.
77 Webcasts/
78 Webcasts as topic/
79 microblog*.mp.
80 micro-blog*.mp.
81 facebook*.mp.
82 Social Media/
83 Social Networking/
84 "information and communication technolog*".mp.
85 ict.mp.
86 etechnolog*.mp.
87 e-technolog*.mp.
88 "health 2.0".mp.
89 "web 2.0".mp.
90 "academia.edu".mp.
91 bebo.mp.
92 dailystrength*.mp.
93 livestrong*.mp.
94 epernicus*.mp.
95 experienceproject*.mp.
96 carepages*.mp.
97 caringbridge*.mp.
98 flickr*.mp.
99 fuelmyblog*.mp.
100 friendica*.mp.
101 friendster*.mp.
102 googleplus*.mp.
103 google plus.mp.
104 hi5.mp.
105 jaiku*.mp.
106 kiwibox*.mp.
107 linkedin*.mp.
108 myopera*.mp.

109 myspace*.mp.
110 netlog*.mp.
111 ning.mp.
112 "ning.com".mp.
113 "www.ning.com".mp.
114 orkut*.mp.
115 pinterest*.mp.
116 researchgate*.mp.
117 sciencestage*.mp.
118 sonico.mp.
119 stumbleupon*.mp.
120 twitter*.mp.
121 wasabi*.mp.
122 "wasabi.com".mp.
123 wellwer*.mp.
124 wooxie*.mp.
125 social awareness*.mp.
126 "aim pages".mp.
127 badoo*.mp.
128 cyworld*.mp.
129 drconnected*.mp.
130 icarecafe*.mp.
131 sanewire*.mp.
132 whoissick*.mp.
133 (social adj2 informatic*).mp.
134 (social adj2 infomatic*).mp.
135 diabetesmine*.mp.
136 "diabetesmine.com".mp.
137 google wave*.mp.
138 "windows live".mp.
139 "live messenger*".mp.
140 "aim messenger*".mp.
141 "yahoo messenger*".mp.
142 "microsoft messenger*".mp.
143 compuserv*.mp.
144 "america online".mp.
145 usenet?.mp.

146 (mobile adj1 technolog*).mp.
147 (technolog* adj1 based adj1 intervention*).mp.
148 mcare.tw.
149 "m-care".tw.
150 "connected care".tw.
151 (health* adj1 inform* adj1 techn*).mp.
152 Web Browser/
153 browser?.mp,kw.
154 (touchscreen* or touch-screen*).tw,kw.
155 (web-bas* or webbas*).mp,kw.
156 Interactive Health Communication.mp.
157 internet communication tool.mp.
158 internet communication.mp.
159 (telemedicine/ or remote consultation/) not teledermatology.mp. not teleradiology.mp. not telepathology.mp. [mp=title, abstract, original title, name of substance word, subject heading word, keyword heading word, protocol supplementary concept word, rare disease supplementary concept word, unique identifier]
160 or/1-159 [MEDLINE Internet Hedge]
161 Chronic Disease/
162 (chronic* adj2 ill*).mp,kw.
163 (chronic* adj disease*).mp,kw.
164 (chronic* adj2 disease*).mp,kw.
165 polypatholog*.mp,kw.
166 poly-patholog*.mp,kw.
167 multiple comorbid*.mp,kw.
168 multiple co-morbid*.mp,kw.
169 (chronic adj2 patholog*).mp,kw.
170 pluri-patholog*.mp,kw.
171 pluripatholog*.mp,kw.
172 multiple longterm condition?.mp,kw.
173 multiple long-term condition?.mp,kw.
174 multiple chronic condition?.mp,kw.
175 (multi-morbid* adj2 condition?).mp,kw.
176 (chronic* adj2 condition?).mp,kw.
177 Comorbidity/
178 (long term adj2 condition?).mp,kw.
179 (longterm adj2 condition?).mp,kw.

180 (chronic adj2 medical adj2 problem*).mp,kw.
181 multimorbid*.mp,kw.
182 (multi-component? adj2 chronic).mp,kw.
183 (multicomponent? adj2 chronic).mp,kw.
184 comorbid*.mp,kw.
185 co-morbid*.mp,kw.
186 multimorbid*.mp,kw.
187 multi-morbid*.mp,kw.
188 (complex* adj3 condition?).mp,kw.
189 (complex adj2 care?).mp,kw.
190 or/161-189 [Chronic Illness or Polypathology or Multiple Morbidity]
191 exp program evaluation/
192 exp program development/
193 exp pilot project/
194 ((patient?? or inpatient?? or outpatient??) adj1 portal?).mp,kw.
195 platform?.mp,kw.
196 tool?.mp,kw.
197 toolkit??.mp,kw.
198 intervention studies/
199 intervention?.mp,kw.
200 prototype?.mp,kw.
201 kiosk??.mp,kw.
202 project?.mp,kw.
203 ((patient? or inpatient? or outpatient?) adj2 program?).mp,kw.
204 ((patient? or inpatient? or outpatient?) adj2 programme?).mp,kw.
205 professional-patient relations/
206 or/191-205 [Intervention or Portal or Tool and related terms]
207 160 and 190 and 206 [Internet and Chronic Illness and Intervention Hedges]
208 limit 207 to (english language and humans)
209 remove duplicates from 208
