# Supplementary material for: Web-Based Tools for Text-Based Patient-Provider Communication in Chronic Conditions: Scoping Review
Source: J Med Internet Res. 2017 Oct 27;19(10):e366. doi: 10.2196/jmir.7987 (PMC5681721; doi:10.2196/jmir.7987)
Supplement: Multimedia Appendix 2 [file jmir_v19i10e366_app2.pdf]

## Appendix 2 EMBASE (Ovid Interface) 1946- Week 1 March 2016

| | |
|---|---|
| 1 | internet:.mp. |
| 2 | online.mp. |
| 3 | on-line.mp. |
| 4 | (virtual not virtual realit:).mp. |
| 5 | world wide web:.mp. |
| 6 | worldwide web:.mp. |
| 7 | www.tw. |
| 8 | web.tw,kw. |
| 9 | web page:.mp. |
| 10 | webpage:.mp. |
| 11 | web site:.mp. |
| 12 | website:.mp. |
| 13 | (portal? and (internet* or online or on-line or computer* or electronic or web or webbased or telehealth or tele-health)).mp. [added March 17 2015] |
| 14 | (portal?? adj3 patient??).mp,kw. [added March 17b 2015] |
| 15 | ehealth.mp. |
| 16 | e-health.mp. |
| 17 | semantic web?.mp. |
| 18 | blog:.mp. |
| 19 | folksonom:.mp. |
| 20 | mashup:.mp. |
| 21 | (social adj2 bookmark:).mp. |
| 22 | (social adj2 book-mark:).mp. |
| 23 | (social adj2 software:).mp. |
| 24 | (sociable adj2 technolog:).mp. |
| 25 | (social adj2 technolog:).mp. |
| 26 | tag cloud:.mp. |
| 27 | (virtual adj2 collabor:).mp. |
| 28 | web api:.mp. |
| 29 | (web adj2 syndicat:).mp. |
| 30 | webcast:.mp. |
| 31 | web-cast:.mp. |
| 32 | web-log:.mp. |
| 33 | weblog:.mp. |
| 34 | wiki:.mp. |
| 35 | social network:.mp. |

36 (social adj2 utilit:).mp.
37 chat.mp.
38 chatroom*.mp.
39 chat-room*.mp.
40 chat group*.mp.
41 chatgroup*.mp.
42 chat techno*.mp.
43 meebo.mp.
44 "second life".mp.
45 secondlife.mp.
46 uhealth.mp.
47 (ubiquit* adj2 comput*).mp.
48 "u-comput*".mp.
49 patientslikeme*.mp.
50 "www.patientslikeme.com".mp.
51 (digital adj2 divid*).mp.
52 (digital adj2 inequit*).mp.
53 "u-health*".mp.
54 "e-health*".mp.
55 epatient*.mp.
56 "e-patient*".mp.
57 edoctor*.mp.
58 e-doctor*.mp.
59 ephysician*.mp.
60 "e-physician*".mp.
61 microblog*.mp.
62 micro-blog*.mp.
63 facebook*.mp.
64 "information and communication technolog*".mp. [added May 2 2012]
65 ict.mp. [added May 2 2012]
66 etechnolog*.mp.
67 e-technolog*.mp.
68 "health 2.0".mp.
69 "web 2.0".mp.
70 "academia.edu".mp.
71 bebo.mp.
72 dailystrength*.mp.

73 livestrong*.mp.
74 epernicus*.mp.
75 experienceproject*.mp.
76 carepages*.mp.
77 caringbridge*.mp.
78 flickr*.mp.
79 fuelmyblog*.mp.
80 friendica*.mp.
81 friendster*.mp.
82 googleplus*.mp.
83 google plus.mp.
84 hi5.mp.
85 jaiku*.mp.
86 kiwibox*.mp.
87 linkedin*.mp.
88 myopera*.mp.
89 myspace*.mp.
90 netlog*.mp.
91 ning.mp.
92 "ning.com".mp.
93 "www.ning.com".mp.
94 orkut*.mp.
95 pinterest*.mp.
96 researchgate*.mp.
97 sciencestage*.mp.
98 sonico.mp.
99 stumbleupon*.mp.
100 wasabi*.mp.
101 "wasabi.com".mp.
102 wellwer*.mp.
103 wooxie*.mp.
104 social awareness*.mp.
10 "aim pages".mp.

5

106 badoo*.mp.

107 cyworld*.mp.

108 drconnected*.mp.

109 icarecafe*.mp.

110 sanewire*.mp.

111 whoissick*.mp.

112 (social adj2 informatic*).mp.

113 (social adj2 infomatic*).mp.

114 diabetesmine*.mp.

115 "diabetesmine.com".mp.

116 google wave*.mp.

117 "windows live".mp.

118 "live messenger*".mp.

119 "aim messenger*".mp.

120 "yahoo messenger*".mp.

121 "microsoft messenger*".mp.

122 compuserv*.mp.

123 "america online".mp.

124 (technolog* adj1 based adj1 intervention*).mp. [June 10 2014]

125 mcare.tw.

126 "m-care".tw.

127 "connected care".tw.

128 (web-bas* or webbas*).mp,kw. [added March 18 2015]

129 internet/

13 exp information science/

| | |
|---|---|
| 0 | |
| 131 | telehealth/ |
| 132 | information system/ |
| 133 | online system/ |
| 134 | computer system/ |
| 135 | telecommunication/ |
| 136 | teleconference/ |
| 137 | computer interface/ |
| 138 | human computer interaction/ |
| 139 | social network/ |
| 140 | social media/ |
| 141 | (health* adj1 communicat* adj1 techn*).mp. |
| 142 | exp computer network/ |
| 143 | webcast/ |
| 144 | interactive health communication.mp. |
| 145 | internet communication tool.mp. |
| 146 | internet communication.mp. |
| 147 | telemedicine/ not teledermatology.mp. not teleradiology.mp. not telepathology.mp. [mp=title, abstract, heading word, drug trade name, original title, device manufacturer, drug manufacturer, device trade name, keyword] |
| 148 | or/1-147 [EMBASE Internet Hedge] |
| 149 | Chronic Disease/ |
| 15 | (chronic* adj2 ill*).mp,kw. |

| | |
|---|---|
| 0 | |
| 151 | (chronic* adj disease*).mp,kw. |
| 152 | (chronic* adj2 disease*).mp,kw. |
| 153 | polypatholog*.mp,kw. |
| 154 | poly-patholog*.mp,kw. |
| 155 | multiple comorbid*.mp,kw. |
| 156 | multiple co-morbid*.mp,kw. |
| 157 | (chronic adj2 patholog*).mp,kw. |
| 158 | pluri-patholog*.mp,kw. |
| 159 | multiple longterm condition?.mp,kw. |
| 160 | multiple long-term condition?.mp,kw. |
| 161 | (multi-morbid* adj2 condition?).mp,kw. |
| 162 | (chronic* adj2 condition?).mp,kw. |
| 163 | Comorbidity/ |
| 164 | (long term adj2 condition?).mp,kw. |
| 165 | (longterm adj2 condition?).mp,kw. |
| 166 | (chronic adj2 medical adj2 problem*).mp,kw. |
| 167 | multimorbid*.mp,kw. |
| 168 | (multi-component? adj2 chronic).mp,kw. |
| 169 | (multicomponent? adj2 chronic).mp,kw. |
| 170 | comorbid*.mp,kw. |

| | |
|---|---|
| 171 | co-morbid*.mp,kw. |
| 172 | multimorbid*.mp,kw. |
| 173 | multi-morbid*.mp,kw. |
| 174 | (complex* adj3 condition?).mp,kw. |
| 175 | (complex adj2 care?).mp,kw. |
| 176 | or/149-175 [EMBASE Chronic Illness or Polypathology or Multiple Morbidity] |
| 177 | exp program evaluation/ |
| 178 | exp program development/ |
| 179 | exp pilot project/ |
| 180 | ((patient?? or inpatient?? or outpatient??) adj1 portal?).mp,kw. |
| 181 | platform?.mp,kw. |
| 182 | tool?.mp,kw. |
| 183 | toolkit??.mp,kw. |
| 184 | intervention studies/ |
| 185 | intervention?.mp,kw. |
| 186 | prototype?.mp,kw. |
| 187 | kiosk??.mp,kw. |
| 188 | project?.mp,kw. |
| 189 | ((patient? or inpatient? or outpatient?) adj2 program?).mp,kw. |
| 190 | ((patient? or inpatient? or outpatient?) adj2 programme?).mp,kw. |
| 19 | or/177-190 [Intervention or Portal or Tool & related terms] |

| | |
|---|---|
| 1 | |
| 192 | 148 and 176 and 191 [EMBASE Internet and Chronic Illness and Intervention Hedges] |
| 193 | limit 192 to (human and english language) |
| 194 | remove duplicates from 193 |
