# Supplementary material for: Web-Based Tools for Text-Based Patient-Provider Communication in Chronic Conditions: Scoping Review
Source: J Med Internet Res. 2017 Oct 27;19(10):e366. doi: 10.2196/jmir.7987 (PMC5681721; doi:10.2196/jmir.7987)
Supplement: Multimedia Appendix 3 [file jmir_v19i10e366_app3.pdf]

Appendix 3 Google Search Strategy

- the first 100 hits of the following strings will be examined for electronic communication tools that meet the specified inclusion and exclusion criteria

Key Terms

1. (electronic | online | web-based | web based | internet | computer-based) (“patient*physician”| “patient*nurse” | “patient*clinician”| “patient* provider”| “patient*pharmacist”) (communication | messaging | contact) (tool | app | application | system | portal | platform | network)

2. (electronic | online | web-based | web based | internet | computer-based) (communication | messaging | contact) (tool | app | application | system | portal | platform | network)

3. (“patient*physician”| “patient*nurse” | “patient*clinician”| “patient* provider”| “patient*pharmacist”) (communication | messaging | contact) (tool | app | application | system | portal | platform | network)

4. (healthcare | physician | provider | clinician | pharmacist | nurse) (eVisits | telehealth | telemedicine)

5. (healthcare | physician | provider | clinician | pharmacist | nurse) (eVisits) (tool | app | application | system | portal | platform | network)

6. (healthcare | physician | provider | clinician | pharmacist | nurse) (patient) (tool | app | application | system | portal | platform | network)
