# Supplementary material for: Web-Based Tools for Text-Based Patient-Provider Communication in Chronic Conditions: Scoping Review
Source: J Med Internet Res. 2017 Oct 27;19(10):e366. doi: 10.2196/jmir.7987 (PMC5681721; doi:10.2196/jmir.7987)
Supplement: Multimedia Appendix 4 [file jmir_v19i10e366_app4.pdf]

Appendix 4 Data extraction form for published literature search

		Reviewer response (not more than 1 sentence)
1.	Citation	First Author:
		Title:
		Journal:
		Year:
5.	What type of publication is this paper?	☐ original research study (observational study, randomized controlled trial etc.)
		☐ editorial/commentary/news and views
		☐ review/meta-analysis
		☐ protocol
		☐ RCT
		☐ Review
		☐ other:______________
12.	Is there a communication component to the tool?	☐ Yes
		☐ No<br>If no, do not continue.
14.	Is the communication component the primary feature or a supplemental feature?	☐ Primary
		☐ Supplemental
16.	What is the primary functioning of the communication component of the tool?	☐ Unstructured (Patient-provider free-form communication)
		☐ Structured (Tailored feedback)
18.	Is the tool findable (website url etc.)?	☐ URL:
		☐ No, further information about the tool could not be found
20.	What is the country of origin?	Please specify:
21.	At what stage of study is the tool?	☐ Development
		☐ Feasibility and piloting
		☐ Evaluation
		☐ Implementation
		☐ Other: _____________
26.	In what context or setting is the tool used?	☐ Healthcare
		☐ Non-healthcare
		☐ Other, please specify: _______________<br>If non-healthcare tools, exclude
		Further description, if needed:
30.	What clinical/disease area is the tool focused on?	☐ Cardiovascular disease
		☐ Cancer

		☐ Stroke
		☐ Chronic respiratory diseases
		☐ Diabetes
		☐ Mental health
		☐ Non-specific
		☐ Other: _______________
38.	What is the primary purpose of the tool?	☐ Patient-healthcare provider communication
		☐ Communication that is not between patients and healthcare providers
		☐ Health information storage, such as an EHR
		☐ Other: _______________
42.	List all other functions of the tool beyond that mentioned in 6.	Description:
43.	Have any published studies been conducted which evaluated this tool?	☐ Yes
		☐ What study design?:
		☐ Randomized controlled trial
		☐ Observational study
		☐ Cohort
		☐ Case-control study
		☐ Non-randomized controlled trial
		☐ Controlled before and after study
		☐ Qualitative study
		☐ Cross-sectional study
53.	If observational study, is it prospective or retrospective?	☐ Prospective
		☐ Retrospective
55.	Is this study a randomized controlled trial?	☐ Yes
		☐ If it was evaluated with the Medical Research Council Framework for Complex Interventions, check phase:
		☐ Phase I
		☐ Phase II
		☐ Phase III
		☐ Phase IV
		☐ No
		Description:
63.	If answered yes to 8, what outcomes were measured?	Description:

64.	What was the study sample size?	Description:
65.	What is the length of follow-up?	Description:
		☐ N/A
67.	What medium does it use?	☐ Website-based
		☐ Cellular Phone-based Secure Messaging Service ("texting")
		☐ Software-based connected by internet
		☐ Web-based app
		☐ Native app
		☐ Hybrid web and native app
		☐ Other: ________________
74.	Is this tool a component of another system/platforms (i.e. EHR, care plan)??	☐ Yes
		☐ If yes, how: ___________
		☐ No
		☐ If no, are there future plans for connectivity:
		☐ Yes, how: ___________
		☐ No
80.	What credentials are used for logging in?	☐ Email address
		☐ Password
		☐ Username
		☐ Banking credentials
		☐ Other: _________________
85.	What form of communication does the tool work by?	☐ Synchronous communication
		☐ Asynchronous communication (end-users do not have to use the tool in real-time)
		☐ Other: _________________
88.	If asynchronous communication, is it time-limited?	☐ Yes
		☐ No
90.	Does the tool engage patients and/or caregivers?	☐ Select all that apply
		☐ Adult patients (default)
		☐ Children
		☐ Family Caregivers
		☐ Parents
		☐ Paid Caregivers

96.	What type of health care provider is intended to use the tool?	Nurse
		Physician
		Physician Assistant
		Pharmacist
		Allied Health Professional (Physiotherapist, Dietician, Social Worker, Psychologist etc.)
		Other: _______________
102.	What is the study context/setting of use?	Academic
		Business (i.e. CVS, Walmart)
		Integrated healthcare organization (i.e. Kaiser Permanente)
		Primary care
		Tertiary care outpatient clinics
		Inpatient hospital units
		Other: ________________
109.	What area of clinical specialty is/are the end-users?	Describe:
110.	Does the tool allow for team-based communication (more than a single health care provider can communicate)?	Yes
		No
		Unclear
113.	Does the patient communicate with their own healthcare provider?	Yes
		No
		Unclear
116.	How is the tool made	Freely available
		Registration required to use
		Payment required
		User pays
		Organizational license
		Other form of monetary revenue: ________
122.	What is the name of the tool?	Description:
