# Supplementary material for: Web-Based Tools for Text-Based Patient-Provider Communication in Chronic Conditions: Scoping Review
Source: J Med Internet Res. 2017 Oct 27;19(10):e366. doi: 10.2196/jmir.7987 (PMC5681721; doi:10.2196/jmir.7987)
Supplement: Multimedia Appendix 5 [file jmir_v19i10e366_app5.pdf]

Appendix 5 Internet data extraction form

		Reviewer response (not more than 1 sentence)
1.	URL	
2.	Type of website	☐ Business
		☐ Healthcare
		☐ Other: _______________
5.	Name of the organization	
6.	Name of the tool	
7.	Tool developer	
8.	Terminology for the platform	
9.	Linked to health record?	☐ Yes
		☐ No
		☐ Unclear
12.	Payment/compensation for healthcare providers?	☐ Yes
		☐ If yes, please specify: _______________
		☐ No
		☐ Unclear
16.	Target population	
17.	Does this tool engage patients and/or caregivers?	☐ Yes
		☐ No
		☐ Unclear
20.	What type of provider is intended to use this tool with the patient?	☐ Doctor
		☐ Nurse
		☐ Other:_____________________
23.	What form of communication does the tool work by?	☐ Synchronous communication
		☐ Asynchronous communication (end-users do not have to use the tool in real-time)
		☐ Other: _________________
26.	If asynchronous communication, is it time-limited?	☐ Yes
		☐ No
		☐ Unclear
29.	Does the tool allow for team-based	☐ Yes

	communication (more than a single health care provider can communicate)?	☐ No
		☐ Unclear
32.	Does this tool allow the patient to communicate with their own healthcare provider?	☐ Yes
		☐ No
		☐ Unclear
35.	Can patients self-register to use the tool?	☐ Yes
		☐ No (prior verification is required)
		☐ Unclear
38.	Has the tool been identified through the published literature?	☐ Yes
		☐ No
40.	Country of origin:	
41.	Year last updated:	
