# Supplementary material for: Web-Based Tools for Text-Based Patient-Provider Communication in Chronic Conditions: Scoping Review
Source: J Med Internet Res. 2017 Oct 27;19(10):e366. doi: 10.2196/jmir.7987 (PMC5681721; doi:10.2196/jmir.7987)
Supplement: Multimedia Appendix 6 [file jmir_v19i10e366_app6.pdf]

Appendix 6
Coding Framework

**Article Characteristics**
**Setting**
Tertiary care- the intervention is evaluated in a tertiary care setting, may be outpatient care (i.e. healthcare provider is a specialist or works in a specialty clinic)
Primary care- the intervention is evaluated in a primary care/community setting (i.e. healthcare provider is in primary care or general practice)
Academic- the intervention is evaluated through an academic setting (i.e. healthcare provider is based out of a university department, such as Behavioral Sciences)
Business- intervention is evaluated in a business context (i.e. through CVS Pharmacy, Walmart)
**Tool Characteristics**
**Medium of communication**
Website- URL accessible via an internet browser (whether mobile or computer-based)
Hybrid website/software application- an intervention which uses a software application and uploads information to the internet, which may have a web-based interface as well
Mobile phone-based native application (i.e. SMS)- an app usable on cell phone
Software- a program installed on a computer's hard drive that may be linked to the internet, but stores information on the computer itself as oppose to online
Email-based application- a tool which makes use of email (i.e. text-based communication exchange which houses data on the server of the sender and on the server of the recipient)
**Functionality**
Asynchronous- non-concurrent communication between patient and healthcare provider
Synchronous- patient and healthcare provider(s) must use the tool at the same time (ie telephone)
**Type of communication**
Unstructured communication (patient-provider free-form)- two-way unstructured text-based dialogue
Structured communication- structured responses or recommendations by a trained end-user (healthcare provider/therapist/research assistant) to information input from the patient
**Intended use**
Lifestyle/behavior modification- altering habits or behaviors to improve patient management of illness
Symptom management- reporting, monitoring or medical treatment related to the care of specific symptoms
Care planning- developing and adherence to protocol for administration and receipt of care and medical procedures
Medication adherence- adherence to prescribed medications
**Evaluation**
**Study design[a]**
Randomized controlled trial- an experimental study in which people are randomly allocated to different interventions
Cohort study- a study in which a defined group of people (the cohort) is followed over time,

to examine associations between different interventions received and subsequent outcomes. A ‘prospective’ cohort study recruits participants before any intervention and follows them into the future. A ‘retrospective’ cohort study identifies subjects from past records describing the interventions received and follows them from the time of those records
Case-control study- a study that compares people with a specific outcome of interest (‘cases’) with people from the same source population but without that outcome (‘controls’), to examine the association between the outcome and prior exposure.
Quasi-experimental/Non-randomized controlled trial- an experimental study in which people are allocated to different interventions using methods that are not random.
Cross-sectional study- a study that collects information on interventions (past or present) and current health outcomes, i.e. restricted to health states, for a group of people at a particular point in time, to examine associations between the outcomes and exposure to interventions
Cost-effectiveness analysis- a study evaluating costs, especially relative to effect or utility of an intervention
Qualitative study- involving a method of data collection comprising interviews and focus groups; data is narrative in nature. Approaches include grounded theory, ethnography, phenomenology
**Stage of evaluation**[b]
Development- identifying evidence base, identifying or developing theory, modelling processes and outcomes
Feasibility/piloting- testing procedures, estimating recruitment and retention, determining sample size
Evaluation- assessing effectiveness, understanding change processes, assessing cost-effectiveness
Implementation- dissemination, surveillance and monitoring, long-term follow-up
**Outcomes**
Health outcomes- clinical data, quality of life or care, healthcare utilization, illness knowledge
Usability[1]- ease of use related outcomes
Usage- how the tool was used, e.g. frequency of use
Costs- medical costs, costs related to implementation or use
Acceptability/Feasibility (collected via questionnaire)[1]- described as feasibility or acceptability measure including satisfaction with implementation, interest, willingness-to-use, attitudes
Experience (open-ended/unstructured responses)- focused on perceptions around the intervention
Clinical- clinical data, quality of life or care, healthcare utilization, knowledge

[a]Adapted from the Cochrane Handbook for Systematic Reviews[2]
[b]Based on the MRC 2008 Framework for the Evaluation of Complex Interventions[3]

References

1. Kushniruk AW, Patel VL, Cimino JJ. Usability testing in medical informatics: cognitive approaches to evaluation of information systems and user interfaces. Proc AMIA Annu Fall Symp. 1997:218-22. PMID: 9357620.
2. Higgins JPT, Green S. Cochrane Handbook for Systematic Reviews of Interventions The Cochrane Collaboration; 2011.
3. Craig P, Dieppe P, Macintyre S, Michie S, Nazareth I, Petticrew M, et al. Developing and evaluating complex interventions: the new Medical Research Council guidance. BMJ. 2008 Sep 29;337:a1655. PMID: 18824488. doi: 10.1136/bmj.a1655.
