# Supplementary material for: Web-Based Tools for Text-Based Patient-Provider Communication in Chronic Conditions: Scoping Review
Source: J Med Internet Res. 2017 Oct 27;19(10):e366. doi: 10.2196/jmir.7987 (PMC5681721; doi:10.2196/jmir.7987)
Supplement: Multimedia Appendix 7 [file jmir_v19i10e366_app7.pdf]

Appendix 7 References by selected tool design and function

	Reference	Article description of tool	Medium/ format of communication	Healthcare provider using the tool	Patient-provider communication flow	Type of communication	Functions in addition to communication?
1.	Armstrong et al. 2015[1]	Online model for follow-up management	Website-based tool	Physician	One-to-one	Asynchronous (not real-time)	Yes
2.	Riipa et al. 2014[2] Riipa et al. 2014[3]	Patient portal	Website-based tool	Physician/ Nurse/care team	Patient-multiple providers	Asynchronous (not real-time)	Yes
3.	Ben-Zeev et al. 2014[4]	Text-messaging	Mobile phone-based application	Social worker	One-to-one	Asynchronous (not real-time)	No
4.	Lau et al. 2014[5]	Patient portal	Website-based tool	Physician/ Nurse/care team	Patient-multiple providers	Asynchronous (not real-time)	Yes
5.	Ryan et al. 2013[6]	Internet-based suite of applications	Website-based tool	Interdisciplinary team of providers	Patient-multiple providers	Asynchronous (not real-time)	Yes
6.	Hsiao et al. 2011[7]	Secure electronic messaging system	Website-based tool	Physician/ Nurse	Patient-multiple providers	Asynchronous (not real-time)	No
7.	van der Meer et al. 2009[8]	Internet-based self-management	Website-based tool	Nurse	One-to-one	Asynchronous (not real-time)	Yes
8.	Siva et al. 2008[9]	Email	Email-based application	Physician	One-to-one	Asynchronous (not real-time)	No
9.	Vernmark et al. 2010[10]	Email therapy	Email-based application	Therapist	One-to-one	Asynchronous (not real-time)	No
10.	Berger et al. 2011[11]	Therapist-guided self-help using email	Email-based application	Therapist	One-to-one	Asynchronous (not real-time)	No
11.	van Bastelaar et al. 2008[12]	Web-based self-help	Website-based	Psychologist	One-to-one	Asynchronous (not real-time)	Yes

12.		Allen et al. 2008[13]	E-coaching	Website-based	Nurse	One-to-one	Asynchronous (not real-time)	Yes
13.		Nilsson et al. 2006[14]	Information and Communication Technology (ICT)	Hybrid web and software application	Nurse	One-to-one	Asynchronous (not real-time)	No
14.		Ross et al. 2004[15]	Patient-accessible online medical record	Website-based	Nurse	One-to-one	Asynchronous (not real-time)	Yes
15.		Jones et al. 2015[16]	Web portal	Website-based	Psychologist	One-to-one	Asynchronous (not real-time)	Yes
16.		Blomfield et al. 2014[17]	E-feedback	Website-based	Nurse	One-to-one	Asynchronous (not real-time)	Yes
17.		van Straten et al. 2014[18]	Guided internet intervention	Website-based	Nurse	One-to-one	Asynchronous (not real-time)	Yes
18.		Wayne et al. 2014[19]	Smartphone-assisted intervention	Mobile phone-based native application	Nurse	One-to-one	Asynchronous (not real-time)	Yes
19.		Barberan-Garcia et al. 2014[20]	Information and Communication Technology (ICT)	Hybrid web and software application	Psychologist	One-to-one	Asynchronous (not real-time)	Yes
20.		Mota Pereira et al. 2014[21]	Facebook	Website-based tool	Physician	One-to-one	Asynchronous (not real-time)	No
21.		Lee et al. 2016[22]	Facebook	Website-based tool	Physician	One-to-one	Asynchronous (not real-time)	No
22.		Nobis et al. 2013[23]	Web-based intervention	Website-based tool	Psychologist	One-to-one	Asynchronous (not real-time)	Yes
23.		Fishman et al. 2013[24] Harris et al. 2009[25] Weppner et al.	Shared medical record	Website-based tool	Pharmacist	One-to-one	Asynchronous (not real-time)	Yes

	2010[26]						
24.	Zhang et al. 2013[27]	Internet of Things technology	Hybrid web and software application	Medical staff	One-to-one	Asynchronous (not real-time)	Yes
25.	Nguyen et al. 2013[28]	Internet-based collaborative self-monitoring	Website-based tool	Nurse	One-to-one	Synchronous (real-time)	Yes
26.	Solomon et al. 2012[29]	Web-based intervention	Website-based tool	Unclear	One-to-one	Asynchronous (not real-time)	Yes
27.	Ahmed et al. 2011[30]	Patient portal	Website-based tool	Nurse	One-to-one	Asynchronous (not real-time)	Yes
28.	Brennan et al. 2010[31]	Technology-enhanced practice	Website-based tool	Nurse	One-to-one	Asynchronous (not real-time)	Yes
29.	Sarkar et al. 2010[32]	Patient portal	Website-based tool	Physician	One-to-one	Asynchronous (not real-time)	Yes
30.	Zickmund et al. 2008[33]	Patient portal	Website-based tool	Physician	One-to-one	Asynchronous (not real-time)	Yes
31.	Bond et al. 2007[34]	Internet-based self-management	Website-based tool	Nurse	One-to-one	Both	Yes
32.	Komives et al. 2005[35]	Patient portal	Website-based tool	Physician	One-to-one	Asynchronous (not real-time)	Yes
33.	Gomez et al. 2002[36]	Internet-based self-management	Website-based tool	Physician Psychologist Social Worker	Patient-multiple providers	Asynchronous (not real-time)	Yes
34.	Huang et al. 2014[37]	Internet-based self-management	Mobile phone-based native application	Unclear	One-to-one	Asynchronous (not real-time)	No
35.	Gulmans et al. 2010[38]	Web-based system for patient-professional and interprofessional	Website-based tool	Physician Physiotherapist School/day care professional	Patient-multiple providers	Asynchronous (not real-time)	Yes

		communication					
36.	Oerlemans et al. 2011[39]	Cognitive Behaviour Therapy with personal digital assistant	Hybrid web and software application	Psychologist	One-to-one	Asynchronous (not real-time)	Yes
37.	Nes et al. 2012[40]	Web-based diaries	Hybrid web and software application	Nurse	One-to-one	Asynchronous (not real-time)	No
38.	Kristjansdottir et al. 2013[41] Kristjansdottir et al. 2013[42] Kristjansdottir et al. 2011[43] Jelin et al. 2012[44]	Web-based self-management	Website-based tool	Nurse or physician	One-to-one	Asynchronous (not real-time)	Yes
39.	McMahon et al. 2005[45] Fonda et al. 2009[46]	Internet-based care management	Website-based tool	Counsellor<br><br>Care Manager	One-to-one	Asynchronous (not real-time)	Yes
40.	Homko et al. 2007[47]	Internet-based telemedicine system	Website-based tool	Unclear	Patient-multiple providers	Asynchronous (not real-time)	Yes
41.	Ralston et al. 2009[48]	Web-based care management	Website-based tool	Care Manager	One-to-one	Asynchronous (not real-time)	Yes
42.	Tang et al. 2013[49]	Personal Health Record	Website-based tool	Nurse	One-to-one	Asynchronous (not real-time)	Yes
43.	Zutz et al. 2007[50]	Virtual cardiac rehabilitation	Website-based tool	Nurse/Dietitian/Exercise specialist	One-to-one	Synchronous (real-time)	Yes
44.	Quinn et al. 2011[51]	Patient web portal	Website-based tool	Counsellor	One-to-one	Asynchronous (not real-time)	Yes
45.	Liebreich et al. 2009[52]	Website and email-linked counselling intervention	Website-based tool	Counsellor	One-to-one	Asynchronous (not real-time)	Yes

46.	Bergmo et al. 2009[53]	Web-based consultations	Hybrid web and software application	Physician	One-to-one	Asynchronous (not real-time)	No
47.	Meglic et al. 2010[54]	Information and Communication Technology (ICT)	Website-based tool	Care Manager	Patient-multiple providers	Asynchronous (not real-time)	Yes

References

1. Armstrong AW, Johnson MA, Lin S, Maverakis E, Fazel N, Liu FT. Patient-centered, direct-access online care for management of atopic dermatitis: a randomized clinical trial. JAMA Dermatol. 2015 Feb;151(2):154-60. PMID: 25338198. doi: 10.1001/jamadermatol.2014.2299.
2. Riippa I, Linna M, Ronkko I. The effect of a patient portal with electronic messaging on patient activation among chronically ill patients: controlled before-and-after study. J Med Internet Res. 2014;16(11):e257. PMID: 25413368.
3. Riippa I, Linna M, Ronkko I, Kroger V. Use of an electronic patient portal among the chronically ill: an observational study. J Med Internet Res. 2014;16(12):e275. PMID: 25488754. doi: 10.2196/jmir.3722.
4. Ben-Zeev D, Kaiser SM, Krzos I. Remote "hovering" with individuals with psychotic disorders and substance use: feasibility, engagement, and therapeutic alliance with a text-messaging mobile interventionist. J Dual Diagn. 2014;10(4):197-203. PMID: 25391277. doi: 10.1080/15504263.2014.962336.
5. Lau M, Campbell H, Tang T, Thompson DJ, Elliott T. Impact of patient use of an online patient portal on diabetes outcomes. Can J Diabetes. 2014 Feb;38(1):17-21. PMID: 24485208. doi: 10.1016/j.jcjd.2013.10.005.
6. Ryan JG, Schwartz R, Jennings T, Fedders M, Vittoria I. Feasibility of an internet-based intervention for improving diabetes outcomes among low-income patients with a high risk for poor diabetes outcomes followed in a community clinic. Diabetes Educ. 2013 May-Jun;39(3):365-75. PMID: 23610181. doi: 10.1177/0145721713484594.
7. Hsiao AL, Bazzy-Asaad A, Tolomeo C, Edmonds D, Belton B, Benin AL. Secure web messaging in a pediatric chronic care clinic: a slow takeoff of "kids' airmail". Pediatrics. 2011 Feb;127(2):e406-13. PMID: 21220392. doi: 10.1542/peds.2010-1086.
8. van der Meer V, Bakker MJ, van den Hout WB, Rabe KF, Sterk PJ, Kievit J, et al. Internet-based self-management plus education compared with usual care in asthma: a randomized trial. Ann Intern Med. 2009 Jul 21;151(2):110-20. PMID: 19620163.
9. Siva C, Smarr KL, Hanson KD, Parikh M, Lawlor K, Ge B. Internet use and e-mail communications between patients and providers: a survey of rheumatology outpatients. J Clin Rheumatol. 2008 Dec;14(6):318-23. PMID: 19033871. doi: 10.1097/RHU.0b013e318190b636.
10. Vernmark K, Lenndin J, Bjarehed J, Carlsson M, Karlsson J, Oberg J, et al. Internet administered guided self-help versus individualized e-mail therapy: A randomized trial of two versions of CBT for major depression. Behav Res Ther. 2010 May;48(5):368-76. PMID: 20152960. doi: 10.1016/j.brat.2010.01.005.
11. Berger T, Hammerli K, Gubser N, Andersson G, Caspar F. Internet-based treatment of depression: a randomized controlled trial comparing guided with unguided self-help. Cogn Behav Ther. 2011;40(4):251-66. PMID: 22060248. doi: 10.1080/16506073.2011.616531.
12. van Bastelaar KM, Pouwer F, Cuijpers P, Twisk JW, Snoek FJ. Web-based cognitive behavioural therapy (W-CBT) for diabetes patients with co-morbid depression: design of a randomised controlled trial. BMC Psychiatry. 2008 Feb 19;8:9. PMID: 18284670. doi: 10.1186/1471-244X-8-9.
13. Allen M, Iezzoni LI, Huang A, Huang L, Leveille SG. Improving patient-clinician communication about chronic conditions: description of an internet-based nurse E-coach intervention. Nurs Res. 2008 Mar-Apr;57(2):107-12. PMID: 18347482.

14. Nilsson C, Ohman M, Soderberg S. Information and communication technology in supporting people with serious chronic illness living at home - an intervention study. J Telemed Telecare. 2006;12(4):198-202. PMID: 16774702. doi: 10.1258/135763306777488807.
15. Ross SE, Moore LA, Earnest MA, Wittevrongel L, Lin CT. Providing a web-based online medical record with electronic communication capabilities to patients with congestive heart failure: randomized trial. J Med Internet Res. 2004 May 14;6(2):e12. PMID: 15249261. doi: 10.2196/jmir.6.2.e12.
16. Jones JB, Weiner JP, Shah NR, Stewart WF. The wired patient: patterns of electronic patient portal use among patients with cardiac disease or diabetes. J Med Internet Res. 2015;17(2):e42. PMID: 25707036. doi: 10.2196/jmir.3157.
17. Blomfield RL, Collins CE, Hutchesson MJ, Young MD, Jensen ME, Callister R, et al. Impact of self-help weight loss resources with or without online support on the dietary intake of overweight and obese men: the SHED-IT randomised controlled trial. Obes Res Clin Pract. 2014 Sep-Oct;8(5):e476-87. PMID: 25263837. doi: 10.1016/j.orcp.2013.09.004.
18. van Straten A, Emmelkamp J, de Wit J, Lancee J, Andersson G, van Someren EJ, et al. Guided Internet-delivered cognitive behavioural treatment for insomnia: a randomized trial. Psychol Med. 2014 May;44(7):1521-32. PMID: 24001364. doi: 10.1017/S0033291713002249.
19. Wayne N, Ritvo P. Smartphone-enabled health coach intervention for people with diabetes from a modest socioeconomic strata community: single-arm longitudinal feasibility study. J Med Internet Res. 2014 Jun 06;16(6):e149. PMID: 24907918. doi: 10.2196/jmir.3180.
20. Barberan-Garcia A, Vogiatzis I, Solberg HS, Vilaro J, Rodriguez DA, Garasen HM, et al. Effects and barriers to deployment of telehealth wellness programs for chronic patients across 3 European countries. Respir Med. 2014 Apr;108(4):628-37. PMID: 24451438. doi: 10.1016/j.rmed.2013.12.006.
21. Mota Pereira J. Facebook enhances antidepressant pharmacotherapy effects. ScientificWorldJournal. 2014;2014:892048. PMID: 24574930. doi: 10.1155/2014/892048.
22. Lee JL, Choudhry NK, Wu AW, Matlin OS, Brennan TA, Shrank WH. Patient Use of Email, Facebook, and Physician Websites to Communicate with Physicians: A National Online Survey of Retail Pharmacy Users. J Gen Intern Med. 2016 Jan;31(1):45-51. PMID: 26105675. doi: 10.1007/s11606-015-3374-7.
23. Nobis S, Lehr D, Ebert DD, Berking M, Heber E, Baumeister H, et al. Efficacy and cost-effectiveness of a web-based intervention with mobile phone support to treat depressive symptoms in adults with diabetes mellitus type 1 and type 2: design of a randomised controlled trial. BMC Psychiatry. 2013 Nov 15;13:306. PMID: 24238346. doi: 10.1186/1471-244X-13-306.
24. Fishman PA, Cook AJ, Anderson ML, Ralston JD, Catz SL, Carrell D, et al. Improving BP control through electronic communications: an economic evaluation. Am J Manag Care. 2013 Sep;19(9):709-16. PMID: 24304254.
25. Harris LT, Haneuse SJ, Martin DP, Ralston JD. Diabetes quality of care and outpatient utilization associated with electronic patient-provider messaging: a cross-sectional analysis. Diabetes Care. 2009 Jul;32(7):1182-7. PMID: 19366959. doi: 10.2337/dc08-1771.
26. Weppner WG, Ralston JD, Koepsell TD, Grothaus LC, Reid RJ, Jordan L, et al. Use of a shared medical record with secure messaging by older patients with diabetes. Diabetes Care. 2010 Nov;33(11):2314-9. PMID: 20739686. doi: 10.2337/dc10-1124.
27. Zhang J, Song YL, Bai CX. MIOTIC study: a prospective, multicenter, randomized study to evaluate the long-term efficacy of mobile phone-based Internet of Things in the management

of patients with stable COPD. Int J Chron Obstruct Pulmon Dis. 2013;8:433-8. PMID: 24082784. doi: 10.2147/COPD.S50205.
28. Nguyen HQ, Donesky D, Reinke LF, Wolpin S, Chyall L, Benditt JO, et al. Internet-based dyspnea self-management support for patients with chronic obstructive pulmonary disease. J Pain Symptom Manage. 2013 Jul;46(1):43-55. PMID: 23073395. doi: 10.1016/j.jpainsymman.2012.06.015.
29. Solomon M, Wagner SL, Goes J. Effects of a Web-based intervention for adults with chronic conditions on patient activation: online randomized controlled trial. J Med Internet Res. 2012 Feb 21;14(1):e32. PMID: 22353433. doi: 10.2196/jmir.1924.
30. Ahmed S, Bartlett SJ, Ernst P, Pare G, Kanter M, Perreault R, et al. Effect of a web-based chronic disease management system on asthma control and health-related quality of life: study protocol for a randomized controlled trial. Trials. 2011 Dec 14;12:260. PMID: 22168530. doi: 10.1186/1745-6215-12-260.
31. Brennan PF, Casper GR, Burke LJ, Johnson KA, Brown R, Valdez RS, et al. Technology-enhanced practice for patients with chronic cardiac disease: home implementation and evaluation. Heart Lung. 2010 Nov-Dec;39(6 Suppl):S34-46. PMID: 21092830. doi: 10.1016/j.hrtlng.2010.09.003.
32. Sarkar U, Karter AJ, Liu JY, Adler NE, Nguyen R, Lopez A, et al. The literacy divide: health literacy and the use of an internet-based patient portal in an integrated health system-results from the diabetes study of northern California (DISTANCE). J Health Commun. 2010;15 Suppl 2:183-96. PMID: 20845203. doi: 10.1080/10810730.2010.499988.
33. Zickmund SL, Hess R, Bryce CL, McTigue K, Olshansky E, Fitzgerald K, et al. Interest in the use of computerized patient portals: role of the provider-patient relationship. J Gen Intern Med. 2008 Jan;23 Suppl 1:20-6. PMID: 18095039. doi: 10.1007/s11606-007-0273-6.
34. Bond GE, Burr R, Wolf FM, Price M, McCurry SM, Teri L. The effects of a web-based intervention on the physical outcomes associated with diabetes among adults age 60 and older: a randomized trial. Diabetes Technol Ther. 2007 Feb;9(1):52-9. PMID: 17316098.
35. Komives EM. Clinician-patient E-mail communication: challenges for reimbursement. N C Med J. 2005 May-Jun;66(3):238-40. PMID: 16130953.
36. Gomez EJ, Caceres C, Lopez D, Del Pozo F. A web-based self-monitoring system for people living with HIV/AIDS. Comput Methods Programs Biomed. 2002 Jul;69(1):75-86. PMID: 12088595.
37. Huang JS, Terrones L, Tompane T, Dillon L, Pian M, Gottschalk M, et al. Preparing adolescents with chronic disease for transition to adult care: a technology program. Pediatrics. 2014 Jun;133(6):e1639-46. PMID: 24843066. doi: 10.1542/peds.2013-2830.
38. Gulmans J, Vollenbroek-Hutten MM, Visser JJ, Nijeweme-d'Hollosy WO, van Gemert-Pijnen JE, van Harten WH. A web-based communication system for integrated care in cerebral palsy: design features, technical feasibility and usability. J Telemed Telecare. 2010;16(7):389-93. PMID: 20736247. doi: 10.1258/jtt.2010.091013.
39. Oerlemans S, van Cranenburgh O, Herremans PJ, Spreeuwenberg P, van Dulmen S. Intervening on cognitions and behavior in irritable bowel syndrome: A feasibility trial using PDAs. J Psychosom Res. 2011 Mar;70(3):267-77. PMID: 21334498. doi: 10.1016/j.jpsychores.2010.09.018.
40. Nes AA, van Dulmen S, Eide E, Finset A, Kristjansdottir OB, Steen IS, et al. The development and feasibility of a web-based intervention with diaries and situational feedback via

smartphone to support self-management in patients with diabetes type 2. Diabetes Res Clin Pract. 2012 Sep;97(3):385-93. PMID: 22578890. doi: 10.1016/j.diabres.2012.04.019.
41. Kristjansdottir OB, Fors EA, Eide E, Finset A, Stensrud TL, van Dulmen S, et al. A smartphone-based intervention with diaries and therapist feedback to reduce catastrophizing and increase functioning in women with chronic widespread pain. part 2: 11-month follow-up results of a randomized trial. J Med Internet Res. 2013 Mar 28;15(3):e72. PMID: 23538392. doi: 10.2196/jmir.2442.
42. Kristjansdottir OB, Fors EA, Eide E, Finset A, Stensrud TL, van Dulmen S, et al. A smartphone-based intervention with diaries and therapist-feedback to reduce catastrophizing and increase functioning in women with chronic widespread pain: randomized controlled trial. J Med Internet Res. 2013 Jan 07;15(1):e5. PMID: 23291270. doi: 10.2196/jmir.2249.
43. Kristjansdottir OB, Fors EA, Eide E, Finset A, van Dulmen S, Wigers SH, et al. Written online situational feedback via mobile phone to support self-management of chronic widespread pain: a usability study of a Web-based intervention. BMC Musculoskelet Disord. 2011 Feb 25;12:51. PMID: 21352516. doi: 10.1186/1471-2474-12-51.
44. Jelin E, Granum V, Eide H. Experiences of a web-based nursing intervention--interviews with women with chronic musculoskeletal pain. Pain Manag Nurs. 2012 Mar;13(1):2-10. PMID: 22341135. doi: 10.1016/j.pmn.2011.08.008.
45. McMahon GT, Gomes HE, Hickson Hohne S, Hu TM, Levine BA, Conlin PR. Web-based care management in patients with poorly controlled diabetes. Diabetes Care. 2005 Jul;28(7):1624-9. PMID: 15983311.
46. Fonda SJ, McMahon GT, Gomes HE, Hickson S, Conlin PR. Changes in diabetes distress related to participation in an internet-based diabetes care management program and glycemic control. J Diabetes Sci Technol. 2009 Jan;3(1):117-24. PMID: 20046656. doi: 10.1177/193229680900300113.
47. Homko CJ, Santamore WP, Whiteman V, Bower M, Berger P, Geifman-Holtzman O, et al. Use of an internet-based telemedicine system to manage underserved women with gestational diabetes mellitus. Diabetes Technol Ther. 2007 Jun;9(3):297-306. PMID: 17561800. doi: 10.1089/dia.2006.0034.
48. Ralston JD, Hirsch IB, Hoath J, Mullen M, Cheadle A, Goldberg HI. Web-based collaborative care for type 2 diabetes: a pilot randomized trial. Diabetes Care. 2009 Feb;32(2):234-9. PMID: 19017773. doi: 10.2337/dc08-1220.
49. Tang PC, Overhage JM, Chan AS, Brown NL, Aghighi B, Entwistle MP, et al. Online disease management of diabetes: engaging and motivating patients online with enhanced resources-diabetes (EMPOWER-D), a randomized controlled trial. J Am Med Inform Assoc. 2013 May 01;20(3):526-34. PMID: 23171659. doi: 10.1136/amiajnl-2012-001263.
50. Zutz A, Ignaszewski A, Bates J, Lear SA. Utilization of the internet to deliver cardiac rehabilitation at a distance: a pilot study. Telemed J E Health. 2007 Jun;13(3):323-30. PMID: 17603835. doi: 10.1089/tmj.2006.0051.
51. Quinn CC, Shardell MD, Terrin ML, Barr EA, Ballew SH, Gruber-Baldini AL. Cluster-randomized trial of a mobile phone personalized behavioral intervention for blood glucose control. Diabetes Care. 2011 Sep;34(9):1934-42. PMID: 21788632.
52. Liebreich T, Plotnikoff RC, Courneya KS, Boule N. Diabetes NetPLAY: A physical activity website and linked email counselling randomized intervention for individuals with type 2 diabetes. Int J Behav Nutr Phys Act. 2009 Mar 27;6:18. PMID: 19327141. doi: 10.1186/1479-5868-6-18.

53. Bergmo TS, Wangberg SC, Schopf TR, Solvoll T. Web-based consultations for parents of children with atopic dermatitis: results of a randomized controlled trial. Acta Paediatr. 2009 Feb;98(2):316-20. PMID: 18795905. doi: 10.1111/j.1651-2227.2008.01033.x.
54. Meglic M, Furlan M, Kuzmanic M, Kozel D, Baraga D, Kuhar I, et al. Feasibility of an eHealth service to support collaborative depression care: results of a pilot study. J Med Internet Res. 2010 Dec 19;12(5):e63. PMID: 21172765. doi: 10.2196/jmir.1510.
